# A Landscape Genetics Approach Reveals Species‐Specific Connectivity Patterns for Stream Insects in Fragmented Habitats

**DOI:** 10.1002/ece3.71084

**Published:** 2025-03-09

**Authors:** Vanessa de Araujo Barbosa, S. Elizabeth Graham, Ian D. Hogg, Brian J. Smith, Angela McGaughran

**Affiliations:** ^1^ School of Science University of Waikato Hamilton New Zealand; ^2^ National Institute of Water and Atmospheric Research—NIWA Hamilton New Zealand; ^3^ Polar Knowledge Canada Canadian High Arctic Research Station Cambridge Bay Nunavut Canada

**Keywords:** aquatic insects, connectivity, dispersal, landscape genetics, population structure

## Abstract

Dispersal is a critical process in ecology and evolution, shaping global biodiversity patterns. In stream habitats, which often exist within diverse and fragmented landscapes, dispersal ensures population connectivity and survival. For aquatic insects in particular, landscape features may significantly influence the degree of genetic connectivity among populations. Thus, understanding connectivity drivers in such populations is essential for the conservation and management of streams. We conducted a landscape genetic study using mitochondrial DNA (mtDNA) and genome‐wide single nucleotide polymorphism (SNP) markers to assess the functional connectivity of stream insects in a fragmented pasture‐dominated landscape. We focused on three species with terrestrial winged adults: the mayfly *Coloburiscus humeralis*, the stonefly *Zelandobius confusus*, and the caddisfly *Hydropsyche fimbriata*. We observed significant spatial genetic structure at larger geographical distances (populations separated by ~30 and 170 km). However, the effects of landscape factors, which were assessed at fine spatial scales, varied among species: for *C. humeralis* SNP data, genetic differentiation was weakly correlated with land cover, suggesting greater population connectivity within stream channels protected by forested riparian zones compared to fragmented streams; for *Z. confusus* , widespread gene flow indicated high dispersal potential across forested and pasture land; while overland dispersal was reduced for *H. fimbriata* (potentially due to local habitat features), this did not seem to hinder broader population connectivity. Our results emphasise the importance of assessing landscape features when evaluating population connectivity in stream riparian zones, which can greatly benefit stream management efforts through an enhanced understanding of connectivity dynamics.

## Graphical abstract

We conducted a landscape genetics study using mtDNA and SNP markers to examine the connectivity and dispersal of three stream insect species, Coloburiscus humeralis, Zelandobius confusus and Hydropsyche fimbriata, in a fragmented, pasture‐dominated landscape on the North Island of New Zealand. Spatial genetic structure with isolation by distance was observed at large scales, while landscape factors such as land cover and topography had a weaker influence on genetic differentiation at finer scales. *Z. confusus* showed high dispersal capacity and low constraints in altered and proximate streams, whereas the short‐distance dispersal of *C. humeralis* and *H. fimbriata* was enhanced in forested stream channels.

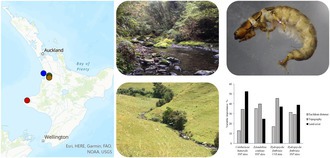

## 1 Introduction

Habitat loss and fragmentation are major threats to biodiversity among natural populations (Fahrig 2003; Lawler et al. 2013). As suitable habitat decreases, the once‐continuous landscape transforms into isolated patches of varying size and connectivity. This phenomenon is especially pronounced in riverine ecosystems, where anthropogenic habitat fragmentation is prevalent and increasing (Reid et al. 2019). Land‐use changes within river catchments with intense agricultural production and urbanisation have had detrimental effects on many freshwater species and on overall ecosystem health, affected mainly by the clearing of native vegetation and decreases in water quality (Dala‐Corte et al. 2016; Fuller et al. 2015). The loss of riparian vegetation in particular has had profound consequences for stream function, altering streambank stability, increasing sedimentation, elevating water temperatures due to reduced shading, and increasing eutrophication, among many other negative effects (Burrell et al. 2014; Hladyz et al. 2011).

The removal of riparian vegetation in streams can have cascading effects on aquatic insect populations that depend on these habitats for foraging, shelter, and reproduction. Forested riparian zones provide corridors and connectivity of suitable microhabitats with shade, protection from strong winds, cooler temperatures, and ideal humidity (Carlson et al. 2016; Collier and Smith 2000). Fragmentation can be a powerful change for riverine habitats and species, and understanding how species successfully disperse through altered patches is vital for the long‐term management of populations (Blanchet et al. 2010; Galic et al. 2013; Fuller et al. 2015). Altered landscapes not only affect dispersal pathways but also restrict movements of individuals, potentially disrupting population connectivity, reducing gene flow, and leading to decreased effective population size and genetic diversity through genetic drift and inbreeding (Pavlova et al. 2017; Schlaepfer et al. 2018).

Stream habitat fragmentation may also have profound effects on local riverine landscapes and species (Blanchet et al. 2010; Galic et al. 2013; Fuller et al. 2015). For stream insects, dispersal and colonisation predominantly follow the stream channel, emphasising the importance of longitudinal connectivity (Petersen et al. 2004; Wiens 2002). Additionally, population genetic studies have highlighted the importance of lateral connectivity in shaping biodiversity patterns for riverine systems (Alp et al. 2012; Geismar et al. 2015; Hughes 2007; Wilcock et al. 2007; Yaegashi et al. 2014), and indicated how various factors can affect aerial dispersal (including topographic or anthropogenic physical barriers, weather, and land cover; Blakely et al. 2006; Parkyn and Smith 2011; Phillipsen and Lytle 2013). Most restoration efforts for fragmented habitats rely on natural recolonisation of the restored habitat via dispersal of individuals from nearby areas (Blakely et al. 2006; Bond and Lake 2003). However, active approaches that specifically facilitate gene flow among populations are essential to ensure such recolonisation (Christie and Knowles 2015). Therefore, a comprehensive understanding of functional population connectivity and dispersal patterns in modified landscapes is a vital aspect of conservation and restoration.

Functional connectivity can be defined as “the degree to which the landscape facilitates or impedes movement along resource patches” (Taylor et al. 1993)—a concept that is both species and landscape‐specific. It also refers to the extent to which populations are able to exchange individuals, maintain genetic diversity and persist over time (Davis et al. 2018; Tischendorf and Fahrig 2000). In streams, the life history and dispersal traits of a particular species, together with the dendritic structure of the stream network and the spatial location of individuals, determine functional connectivity (Hughes et al. 2009). Accordingly, the pattern and scale of population genetic structure varies among taxa. Gene flow may range from high levels among populations for species with high dispersal capacity and no specific habitat requirements (leading to low genetic structure), to low levels for those with low dispersal potential and/or particular habitat requirements (creating high genetic structure) (Finn et al. 2007; Hughes et al. 2009).

Functional connectivity can be assessed by investigating the correlation between population genetic differentiation and landscape and/or environmental features, known as ‘landscape genetics’ (Manel and Holderegger 2013; Manel et al. 2003; Spear et al. 2010). Isolation By Distance (IBD) is commonly used as a null model, where genetic differentiation is explained solely by geographic distance, assuming no influence of landscape heterogeneity. In contrast, Isolation By Resistance (IBR) incorporates the effects of landscape features on connectivity by examining whether specific portions of the landscape act as facilitators or barriers to gene flow (IBR; McRae 2006; Shah and McRae 2008). Additionally, other mechanisms, such as Isolation By Barrier (e.g., discrete physical or anthropogenic barriers) and Isolation By Environment (e.g., environmental gradients or niche divergence), also demonstrate how landscapes can influence genetic structure (Ringbauer et al. 2018; Wang and Bradburd 2014). While the application of landscape genetics methods to dispersal studies often focuses on terrestrial organisms, aquatic insect research has traditionally relied on IBD models (Grummer et al. 2019). However, studies applying landscape genetic/genomic approaches demonstrate that natural and anthropogenic features, such as barriers and environmental gradients, can shape population connectivity beyond the effects of geographic distance alone (Galic et al. 2013; Phillipsen and Lytle 2013; Keller et al. 2012; Polato et al. 2017).

Here, we conducted a fine‐scale landscape genetic analysis in a native forest landscape fragmented by agriculture and dominated by open pasture, using three different endemic New Zealand stream insect species with winged adult stages: the mayfly *Coloburiscus humeralis* (Ephemeroptera: Coloburiscidae), the stonefly *Zelandobius confusus* (Plecoptera: Gripopterygidae), and the caddisfly *Hydropsyche fimbriata* (Trichoptera: Hydropsychidae). We used mitochondrial DNA (mtDNA) and genome‐wide single nucleotide polymorphism (SNP) markers to: (1) assess functional connectivity between populations by examining their spatial genetic structure; (2) test the effect of pure space (i.e., Euclidean distance; IBD) versus the intervening landscape (IBR) on population connectivity among habitats; and (3) identify landscape elements (i.e., topography and land cover) that inhibit or facilitate dispersal and gene flow. For the three studied species, we predicted that a pattern of IBD would be evident at larger spatial scales (i.e., among populations from different mountain regions). At finer spatial scales, we predicted that open pasture would act as a barrier to dispersal within and among streams. Based on our results, we identify species‐specific patterns of dispersal and connectivity and discuss the associated implications for management and restoration planning.

## 2 Methods

### 2.1 Study Area

**FIGURE 1:**
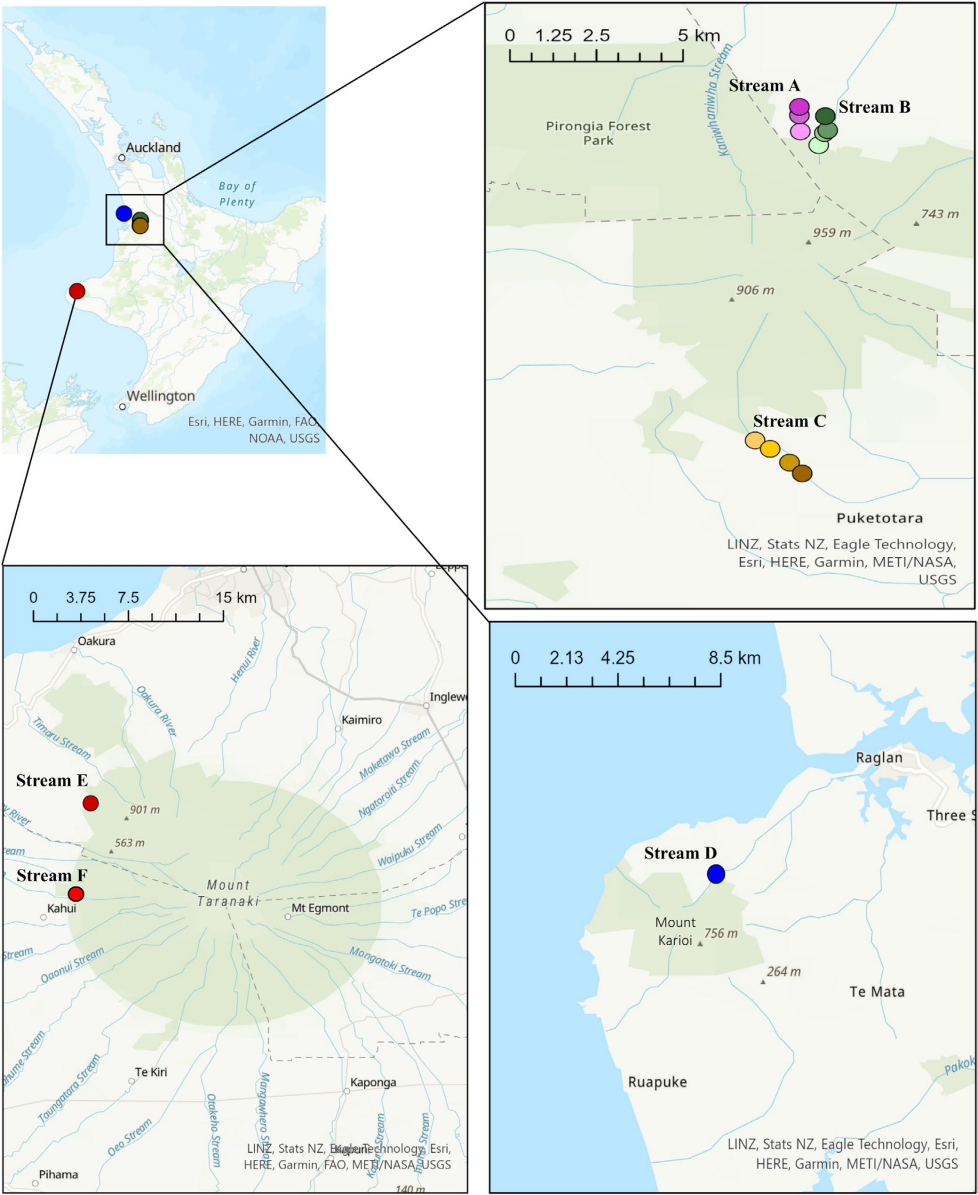
Location of 13 sampling sites distributed in five streams in the North Island of New Zealand. Dots show the smaller detail of the sampling location within the stream. Further locality data are given in Table S1.

This research was conducted in first and second‐order perennial, stony‐bottom streams primarily in the southeast of Mount Pirongia (North Island, New Zealand), covering a spatial area of ~11 km. Additional mountain regions were also included in our broader spatial analysis: Wainui Stream in Mount Karioi (~30 km away from Mount Pirongia) and Katikara Stream and Patea stream in Mount Taranaki (~170 km away from Mount Pirongia) (Figure 1). All three regions are characterised by their intensive deforestation since human colonisation (~1000 years ago), and agricultural land use is one of the main drivers of forest loss (Brockerhoff et al. 2010). Thus, the landscape structure consists of large and small forest fragments surrounded by intense agricultural production (predominantly dairy and sheep farmland) with many resident streams and rivers flowing through native forest before entering agricultural landscapes.

### 2.2 Study Species

We selected three species from the EPT fauna in New Zealand, a group of aquatic insects comprising Ephemeroptera (mayflies), Plecoptera (stoneflies), and Trichoptera (caddisflies). These insects are widely recognised for their importance as indicators of freshwater ecosystem health, as they are sensitive to changes in water quality and environmental stressors (Boothroyd and Stark 2000; Jacobus et al. 2019). The mayfly *C. humeralis* is widely distributed in New Zealand's fast‐flowing streams and rivers, with nymphs commonly inhabiting the underside of stones, predominantly in riffles during its lifespan of 12–27 months (Harding and Winterbourn 1993). The stonefly *Z. confusus* is widely distributed throughout New Zealand, and nymphs are often found in accumulated leaf packs or woody debris within streams. The nymph stage of *Z. confusus* lasts 9–12 months (McLellan 1993). The caddisfly *H. fimbriata* is restricted to the North Island of New Zealand, where larvae can be mostly found in small, stony, forested streams during its lifespan of 9–12 months (Cowley 1978; Winterbourn et al. 2000). All three species have a winged adult stage (living for a few days to weeks; Collier and Smith 2000; Smith B., unpublished data), during which overland dispersal may occur.

### 2.3 Insect Collection

Within the main study area (Mount Pirongia), we sampled 11 sites from three streams located in two neighbouring catchments (Figure 1). Sampling sites were selected based on their land cover features (fully riparian forested, patches of forest upstream, pasture covered downstream), ease of access, and presence of the studied species. Samples were collected from 3 to 4 sites per stream at intervals of at least 490 m (Table S1). All streams were perennial with a consistent flow of water, boulder and cobble bottoms, and little fine sediment deposition. All sampling sites were located at the base of the mountain at a similar elevation (Figure S2), but with different land cover (native forest, forest fragments, pasture; Figure S3). Tawhitiwhiti Stream (Stream A) included one forested site (upstream) and two sites covered by pasture; Te Pahu stream (Stream B) included one forested site, one forest fragment, one pasture site, and one restored (riparian planted) site; Ngakoaohia Stream (Stream C) was mostly confined by steep limestone walls and was fully covered by indigenous forest in the riparian zone. The three additional sampling sites, Mount Karioi (Wainui Stream—Site D1) and Mount Taranaki (Katikara Stream, Site E1 and Patea Stream, Site F1) sites were both fully forested upstream of the sampling location (Table S1).

Mayfly and stonefly nymphs and caddisfly larvae were collected using a kick‐net or hand‐picked from the substrate and immediately preserved in 95% ethanol. Collection occurred over different time points, with samples from the Pirongia sites (the main study area) mostly collected in December 2017 and August/September 2018, and those from Karioi (D1 site) taken in April 2019. Additionally, samples from Taranaki (E1 and F1 sites) were collected in August and December 2019. Individuals were collected from each of the 13 localities. The mean number of samples collected for each species across all 11 Mount Pirongia sites was 55, 49 and 34 for *C. humeralis* , *H. fimbriata* and *Z. confusus* , respectively. The total number of individuals collected in Mount Karioi—Wainui Stream was 30 ( *C. humeralis* ), 31 ( *H. fimbriata* ) and 6 ( *Z. confusus* ), and in Mount Taranaki streams was *C. humeralis* = 18, *H. fimbriata* = 21 and *Z. confusus* = 10. Nymphs of *Z. confusus* were identified (McLellan 1993), and *C. humeralis* and *H. fimbriata* were confirmed, using Winterbourn et al. (2000). The nomenclature of *H. fimbriata* follows Geraci et al. (2010). Samples were then transferred to vials with fresh 95% ethanol and stored at −20°C. The left rear leg was dissected from each individual using sterilised forceps and added to a single well of a 96‐well PCR plate for DNA extraction.

### 2.4 DNA Extraction, mtDNA and SNP Sequencing

Genomic DNA was re‐used or re‐extracted from 286 individuals reported in a previous mtDNA data analysis (Barbosa et al. 2022) and newly extracted from an additional 372 individuals, resulting in a final dataset of 658 individuals for SNP sequencing. Per species, the total number of individuals for which DNA was extracted was *C. humeralis* (mtDNA *n* = 97, SNP *n* = 208), *Z. confusus* (mtDNA *n* = 114, SNP *n* = 160) *and H. fimbriata* (mtDNA *n* = 107, SNP *n* = 293).

DNA extraction and amplification of mtDNA cytochrome *c* oxidase subunit I (COI) gene fragments was conducted at the Canadian Centre for DNA Barcoding following standard protocols (see Ivanova et al. 2006). In brief, DNA was extracted following the AcroPrepTM PALL Glass Fibre plate method using a total mix of 5 mL insect lysis buffer (0.5 mL of Proteinase K, 20 mg/mL per 96‐well plate). A 658 bp region of the COI gene was PCR amplified using the primer pair LepF1 and LepR1 (Hebert et al. 2004; Wilson 2012) and 5 μL of the DNA extraction product. PCR thermal cycling conditions were: initial denaturation of samples at 94°C for 1 min, followed by five cycles of 94°C for 30 s, 48°C for 1.5 min and 72°C for 1 min. This was followed by 35 cycles of 94°C for 30 s, 52°C for 1 min and 72°C for 1 min, with a final extension of 72°C for 10 min. PCR products were cleaned using Sephadex and then sequenced using an ABI3730xl DNA analyser.

DNA extraction, sequencing and SNP genotyping were performed by Diversity Array Technology Pty Ltd. (DarTseq), Canberra, Australia. DNA samples were digested using the PstI‐SphI restriction enzyme pair after a pilot study was performed to identify the enzyme combination most suitable for genome complexity reduction in the target species. The PstI‐compatible forward adapter included the Illumina flow cell attachment sequence, sequencing primer, and a barcode for sample identification within pooled libraries. The reverse adapter contained the Illumina flow cell attachment region and the SphI‐compatible overhang sequence. PstI‐SphI ligated fragments were amplified by adapter‐mediated PCR as follows: initial denaturation at 94°C for 1 min followed by 30 cycles of denaturation at 94°C for 20 s, annealing at 58°C for 30 s and extension at 72°C for 45 s, and an additional final extension step at 72°C for 7 min. Following PCR amplification, equimolar amounts of amplification product for each sample were pooled before 77 cycles of single‐read next‐generation sequencing on the HiSeq2500 (Illumina) platform. Raw reads were processed using a proprietary DarT analytical pipeline that included filtering and variant calling and the generation of final genotypes. Two technical replicates of each DNA sample were genotyped to calculate the reproducibility of the marker data. Finally, SNPs that were polymorphic across samples for each species were obtained from DarTseq, with markers scored as binary data: ‘1’ for presence, ‘0’ for absence, and ‘−’ for failure to score.

### 2.5 Spatial Population Structure Analysis

#### 2.5.1 mtDNA COI Data

Haplotype networks were constructed, assigning individuals to their respective sampling site locations, using the packages ape v.5.5 (Paradis and Schliep 2018) and pegas v.1.0 (Paradis 2010) in R v.4.1.2 (R Core Team 2020). We partitioned total genetic variation into geographic hierarchies using independent analysis of molecular variance (AMOVA) analyses in ARLEQUIN v. 3.5 (Excoffier and Lischer 2010) with 10,000 permutations. For this analysis, populations were partitioned into: “between mountain regions”, “between neighbouring catchments”, “between adjacent streams”, “within Stream A”, “within Stream B” and “within Stream C”. Simple Mantel tests were conducted to assess IBD using both genetic distances (raw *F*_ST_, and linearised *F*_ST_: *F*_ST_/(1—*F*_ST_), as per the suggestion of Rousset (1997) and geographic distances (Euclidean distance and shortest waterway distance). Testing these two types of distances allows determination of how genetic differentiation between populations is either influenced by straight‐line geographic distance (ignoring stream network features) or by the shortest waterway distance (i.e., considering natural corridors to provide a more ecologically relevant measure of connectivity). Mantel tests were conducted in three hierarchical groups: “among all sampling sites”, “among sites within Mount Pirongia” and “among sites within each stream” using the mantel function in the R package vegan v. 2.5‐7 (Oksanen et al. 2020), with *p*‐values estimated from 10,000 permutations. The multi‐level Mantel test analysis can indicate how closely related populations within the same stream reach and between different streams are, considering the potential dispersal distance of the studied species. Analysing all sampling sites together allows for a comprehensive assessment of genetic structure across the entire study area. Due to an absence of sequence data for *H. fimbriata* at Mount Karioi and Taranaki sites, analyses of spatial population genetic structure on this species were only calculated for sites within the Pirongia region.

#### 2.5.2 SNP Data

Data quality control and filtering were performed using the R package DartR v. 1.9.9.1 (Gruber et al. 2018). After testing different parameter combinations (e.g., missing data 5%, 10%, 20%, and minor allele frequency—MAF < 0.02, < 0.05) that showed no significant differences in the downstream population genetic data analysis (data not shown), we proceeded with the following parameter settings for SNP filtering: SNP sites with > 20% missing data, MAF < 0.05, and/or an unknown position were removed.

Pairwise population genetic differentiation was estimated using *F*_ST_ (Weir and Cockerham 1984) and Nei's distance (Nei 1972), using the R package StAMPP v. 1.6.2 (Pembleton et al. 2013). Visualisation of population structure was performed using Principal Coordinates Analysis (PCoA) in DartR with associated functions from the ADEGENET package v. 2.1.4 (Jombart 2008). Separate PCoA plots were also generated for each of the three streams to investigate whether the structure between populations within streams varied with riparian land cover. fastSTRUCTURE v. 1.0 (Raj et al. 2014) was used to identify admixture proportions among individuals and populations, testing 1–13 genetic clusters (depending on the availability of data from each sampling site/population) via the *K* parameter. Five replicates were run for each *K* value using a simple prior. Results for each *K* value were visualised using the distruct.py script, and model complexity was chosen using the chooseK.py script, both associated with the fastSTRUCTURE program. To aid visualisation of population structure, we investigated the association of the resulting genetic clusters to the type of riparian land cover at each sampling site using PCoA. AMOVA was performed with the same geographic hierarchies as described for the COI data, using the poppr v. 2.9.3 package (Kamvar et al. 2014) in R. Mantel tests were performed on the SNP data as described for the COI data (above).

### 2.6 Fine‐Scale Landscape Genetics Analysis for Mount Pirongia Populations

#### 2.6.1 Landscape Data and Rasters

We calculated two connectivity indices to test for IBR: land cover resistance and topographic resistance. For the land cover resistance (LCR), we used the land cover database (10 m resolution) for mainland New Zealand (LCDB v. 5.0) from the land resource information system (LRIS). We chose the updated map from 2018, as this was the closest time to our sampling period. The original shapefiles were processed to raster maps using Geopandas v. 0.10.2 (Jordahl et al. 2020) and Geocube v. 0.1.2 libraries in Python v. 2.7 (Van Rossum and Drake Jr. 1995), and the data classes of interest were filtered. This included seven forest land cover‐related classes and four agri‐related grassland classes predominantly used for livestock grazing, which were used to build a final raster with the classes ‘forest’ and ‘pasture’ (Figure S3), and an analysis of forest versus pasture was used to test whether open pasture was a barrier to dispersal. We selected resistance costs based on the assessment of a range of resistance values (Table 2) (Richardson 2012), as relying on expert opinion alone can introduce subjectivity (Spear et al. 2010). This enabled us to estimate the potential resistance of either vegetation or pasture to connectivity (and therefore influence on dispersal) for each of the three insects. Overall, six land cover rasters were generated using three different resistance values assigned to each of the two categorical landscape variables.

For topographic resistance, we used an 8 m resolution digital elevation model (DEM) sourced from the land information New Zealand (LINZ) to calculate a topographic complexity raster based on the topographic slope values (Figure S2), using the GDAL library v. 3.4.1 (GDAL/OGR Contributors 2020). Slope values were further interpolated to the same geographical points as represented by the LCR data. ‘Slope’ was chosen as a topographic variable based on the potential preference for adult flight within stream valley corridors confined by steep sloping sides that may also inhibit lateral movement (Hughes et al. 1999; Phillipsen et al. 2015; Winterbourn 2007). As suggested by Spear et al. (2010), we used the raw slope values of the map pixels as a continuous variable to assign resistance values to our maps, with values varying from 1 (indicating flat areas and lowest resistance) to 60 (deepest slope and highest resistance), rather than assigning relative costs. This enabled us to test the prediction that higher slopes resulted in lower levels of gene flow or higher genetic differentiation. The final resistance raster was transformed to a 10 m resolution (to be compatible with the land cover raster) for subsequent analysis.

#### 2.6.2 Estimating Resistance Distances From Landscape Rasters

We calculated resistance distances between populations for the topographic raster and each of the potential six land cover resistance rasters by: (1) estimating pairwise least‐cost pathways between populations using the genleastcost function in the R package PopGenReport v. 3.0.4 (Adamack and Gruber 2014), which calculates the shortest distance between pairs of populations based on the given landscape resistance; and (2) using CIRCUITSCAPE v. 4.0 (McRae 2006), which uses information on how current flows across the resistance landscape to estimate the resistance distance between focal points. This enabled the estimation of the resistance of the landscape to gene flow between pairs of populations while assuming that the movement of individuals is not optimal across the landscape so that multiple paths contribute to effective dispersal.

#### 2.6.3 Resistance Model Optimisation and Exclusion of Spurious Variables

We next conducted an exploratory analysis using simple Mantel tests to optimise our resistance model. Each pairwise resistance distance from the least‐cost and CIRCUITSCAPE analyses was correlated with genetic distance (*F*_ST_) to compare the fit and significance of the relationship for each of the competing models. Final land cover resistance costs and variables were selected based on the results of the simple Mantel tests: for each species, the land cover resistance representation with the highest correlation coefficient was chosen, and any uncorrelated variables were excluded from further analysis. We also used the Mantel test results to choose between least‐cost or CIRCUITSCAPE distance matrices, with the highest coefficient retained.

#### 2.6.4 Assessing Landscape Variables Importance

To assess the relative importance of different landscape variables identified in the exploratory Mantel analysis, we used multiple regression on distance matrices (MRM; Balkenhol et al. 2009; Wang 2013), accounting for the inherent spatial structure in the data and enabling the simultaneous use of distinct distance matrices, allowing inferences to be made at the level of individual landscape variables (Lichstein 2007). We conducted MRM analysis on seven potential models using three landscape variables (Euclidean distance, topographic slope and land cover‐forest and pasture) to investigate the importance of geographic distance (IBD) versus landscape resistance (IBR) on shaping spatial genetic structure, namely whether the degree of genetic variation was: (1) solely due to spatial influence (i.e., IBD Euclidean distances); (2) purely attributable to the heterogeneous landscape (i.e., IBR, represented by resistance distances from topographic and land cover rasters) or (3) influenced by a shared component, which cannot be separated into purely spatial IBD versus purely landscape IBR contributions (e.g., because of correlations between Euclidean and resistance distances). Landscape factors can influence genetic differentiation to varying degrees; thus, multiple regression analysis was used to capture their combined effects on genetic variation.

Analyses were conducted using the MRM function in the R package ECODIST v. 2.0.7 (Goslee and Urban 2007). Because each effective distance was used for multiple statistical tests, inferences on both Mantel tests and MRM were based on Bonferroni‐corrected *p*‐values for multiple comparisons. We assessed multicollinearity in the full models using variance inflation factors (VIF) with the vif function in the R package car v. 3.0‐12 (Weisberg and Fox 2011). All variables showed VIF scores < 6 (Table S5) and were therefore retained in the candidate models analysed (Dormann et al. 2013; Zuur et al. 2010). Next, we used hierarchical partitioning to assess the relative importance of the three landscape variables for predicting spatial genetic structure. This approach determines which of the various independent variables in the model has the strongest influence on the response variable by calculating the average variable's contribution to the response variable over all possible combinations of the independent variables (Murray and Conner 2009).

## 3 Results

### 3.1 Spatial Genetic Structure

Mantel test outcomes based on raw and linearised *F*_ST_ were qualitatively very similar, as were results considering either waterway or Euclidean distance; thus, we present only the latter. Mantel tests conducted within each stream of Mount Pirongia revealed non‐significant results for all three studied species and, as such, are not presented here. Detailed results are shown in Table S2.

#### 3.1.1 Coloburiscus humeralis

**FIGURE 2:**
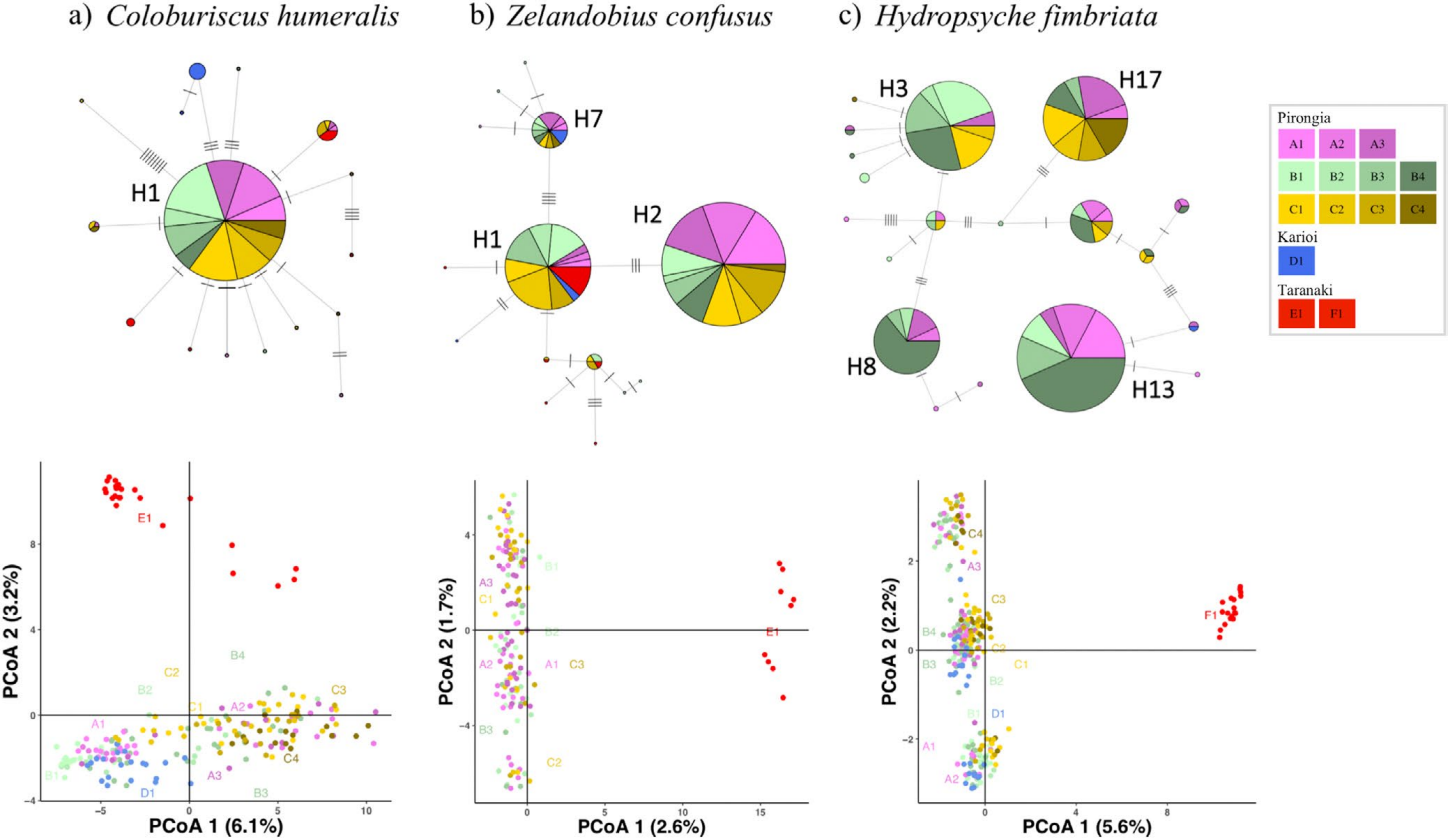
Genetic relationships among individuals and sampling locations for each species across the three regions and streams: Pirongia (stream A, stream B and stream C), Karioi (stream D) and Taranaki (Stream E and Stream F). Individuals are colour‐coded according to collection locality and population label. Above: Haplotype network based on COI sequence data. Each pie chart represents a single haplotype, with the size proportional to the frequency of individuals containing that particular haplotype at each site where samples were collected. Dashes indicate missing mutational steps. Below: Principal Coordinate Analysis (PCoA) based on orthogonal transformation of SNP data. The percentage of variation explained by each principal coordinate is indicated on the axes.

A total of 97 *C. humeralis* individuals with COI sequence data was analysed. The haplotype network consisted of 16 haplotypes, with a few derived haplotypes (including 11 singletons) connecting to a centrally located dominant haplotype (H1). Haplotype H1 was found in 59 individuals located only in Pirongia. Two haplotypes were restricted to the Karioi sampling site, whereas most of the individuals from Taranaki shared the same haplotype with individuals from Pirongia (Figure 2). Global *F*_ST_ revealed high genetic differentiation among all sampling sites (0.428, *p* < 0.001). AMOVA across multiple geographic hierarchies indicated significant differentiation only at the broadest spatial scale (among mountain regions; *F*_ST_ = 0.675, *p* < 0.05; Table 1). The Mantel test provided no evidence for a correlation between Euclidean distance and genetic differentiation across all sampling sites (*r* = 0.009, *p* = 0.157; Figure S1).

**TABLE 1:** Hierarchical analysis of molecular variance (AMOVA) from both markers for each species.

Hierarchical level	*Coloburiscus humeralis* (mayfly)				*Zelandobius confusus* (stonefly)				*Hydropsyche fimbriata* (caddisfly)			
	COI data		SNP data		COI data		SNP data		COI data		SNP data	
	Variance %	*F*_ST_	Variance %	*F*_ST_	Variance %	*F*_ST_	Variance %	*F*_ST_	Variance %	*F*_ST_	Variance %	*F*_ST_
Among all sites	42.85	0.428***	7.40	0.074*	10.24	0.102**	4.83	0.048*	9.23	0.092**	6.97	0.069**
Within all sites	57.15		92.60		89.76		95.16		90.77		93.03	
Across large spatial scales												
Among mountain regions	67.58	0.675*	6.08	0.060*	21.45	0.214*	1.60	0.016*	—	—	18.01	0.180**
Pirongia (main study area)												
Between neighbouring catchments	0.90	0.009	2.50	0.025*	0.10	0.001	4.42	0.044*	14.23	0.142**	1.21	0.012*
Between adjacent streams	0.94	0.009	1.27	0.012*	9.21	0.092	0.12	0.001	0.00	0.000	0.39	0.003
Within stream A	0.00	0.000	4.83	0.048*	0.00	0.000	0.35	0.003	12.01	0.120*	0.00	0.000
Within stream B	6.10	0.061	2.88	0.028*	5.79	0.057	0.35	0.003	0.74	0.007	0.56	0.005
Within stream C	4.06	0.040	0.69	0.006	0.00	0.000	0.42	0.000	0.00	0.000	0.17	0.001

* *p* < 0.05.

** *p* < 0.01.

*** *p* < 0.001—indicates that values could not be obtained due to sample size constraints.

The filtered SNP data set consisted of 4609 SNPs (1.09% missing data) from 208 individuals (Table S3). AMOVA analysis revealed low but significant differentiation among all sampling sites (*F*_ST_ = 0.074, *p* < 0.001, 7.4% variance) and across all spatial scales (Table 1), with the highest *F*_ST_ detected among mountain regions (*F*_ST_ = 0.060, *p* < 0.05) and the lowest detected within Stream C (*F*_ST_ = 0.006, *p* < 0.05, 7.4%). This result contrasted with the COI data, where hierarchical AMOVA analysis only detected significant genetic differentiation among regions. Pairwise *F*_ST_ values were slightly higher than pairwise Nei's D values, but both parameters showed similar patterns of divergence across pairs of populations (Table S4). The highest pairwise *F*_ST_ value was 0.121 (B1/E1), which reflected geographic distance (170.8 km). Contrasting with the COI results, the SNP data indicated IBD within the overall study area (Euclidean distance, *r* = 0.689, *p* = 0.001; Figure S1).

**FIGURE 3:**
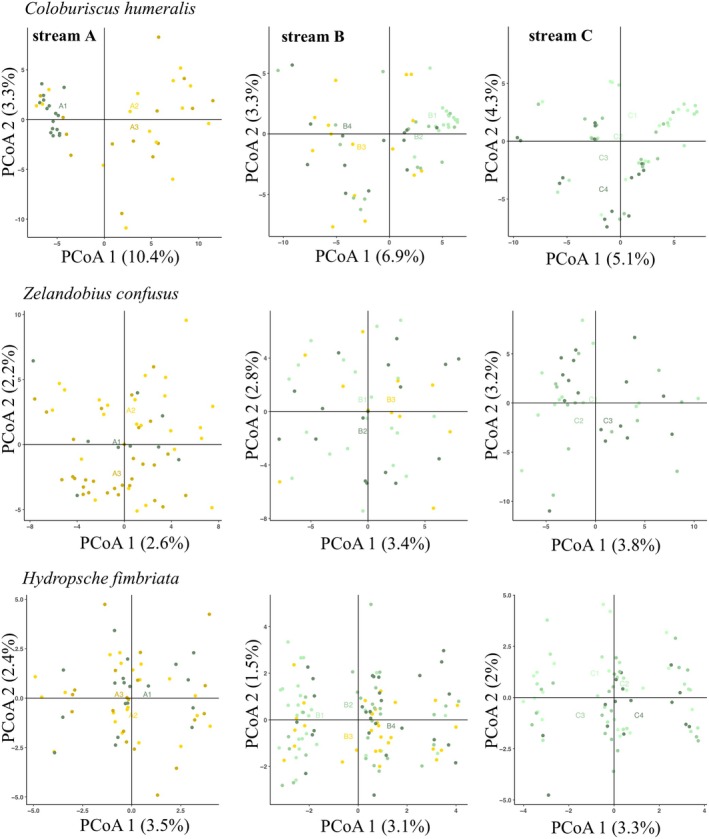
Two‐dimensional plots of Principal Coordinate Analyses (PCoA) based on orthogonal transformation of SNP data for *Coloburiscus humeralis* (mayfly), *Zelandobius confusus* (stonefly), and *Hydropsyche fimbriata* (caddisfly). Data were analysed for each stream in Pirongia separately. Individuals are colour‐coded according to the land cover type (forest × pasture) of each collection locality: Shades of green indicate sampling sites fully covered by riparian forest, and shades of yellow indicate sampling sites covered by pasture open land. The percentage of variation explained by each principal coordinate is indicated on the axes.

In the PCoA, the two principal coordinates explained 6.1% and 2.5% of the total genetic variation (Figure 2). E1 (Taranaki) clustered separately from the remaining populations, and D1 (Karioi) clustered together with most individuals from Streams A and B in Pirongia, and separately from Stream C. When examining clustering patterns associated with land cover type (forested × pasture) in individual streams, the PCoA showed that individuals from forested sites (A1 and B1 in Figure 3) tended to cluster together in fragmented streams. In the fully forested stream C, no clustering associated with a specific sampling site was observed.

**FIGURE 4:**
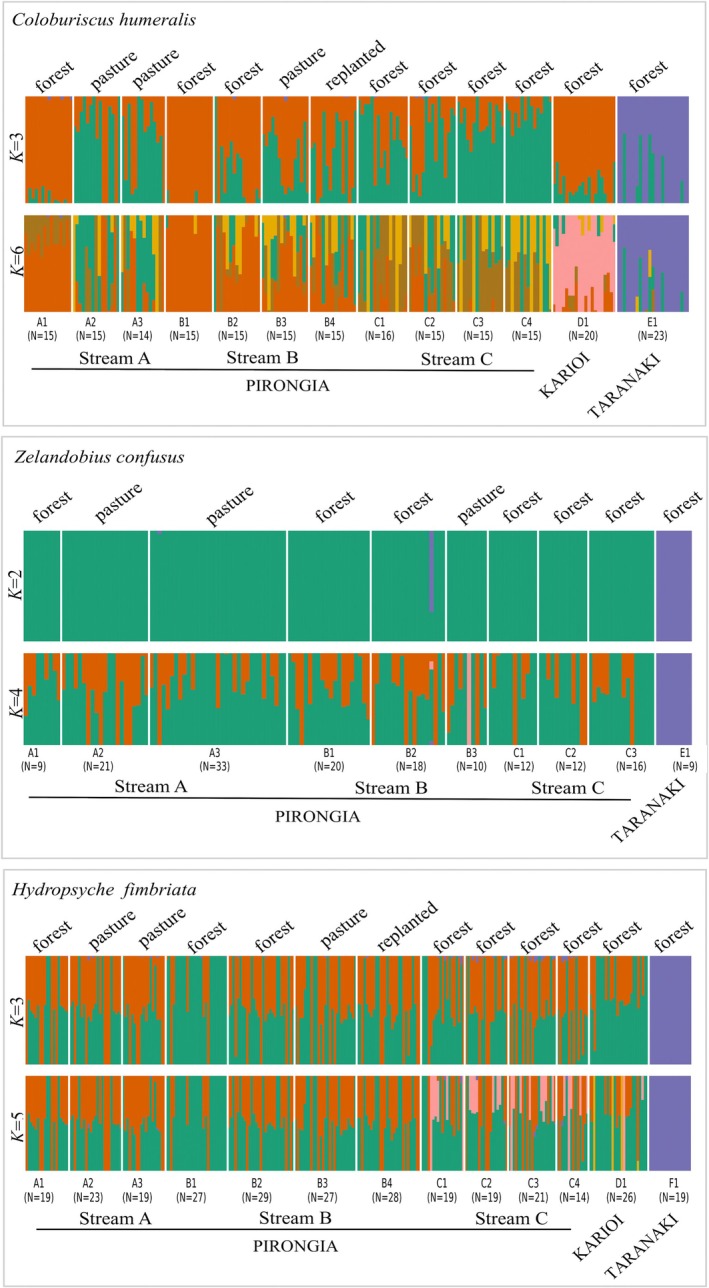
fastSTRUCTURE bar plots representing population structure and admixture coefficients for each of the three species. Colours in each panel represent the assignment of individuals to each genetic cluster. Single bars with > 1 colour indicate admixture of that particular individual (i.e., sharing of genetic ancestry across more than one genetic cluster). Sampling sites are designated by their site codes and locations, and riparian land cover type is indicated for each site.

**FIGURE 5:**
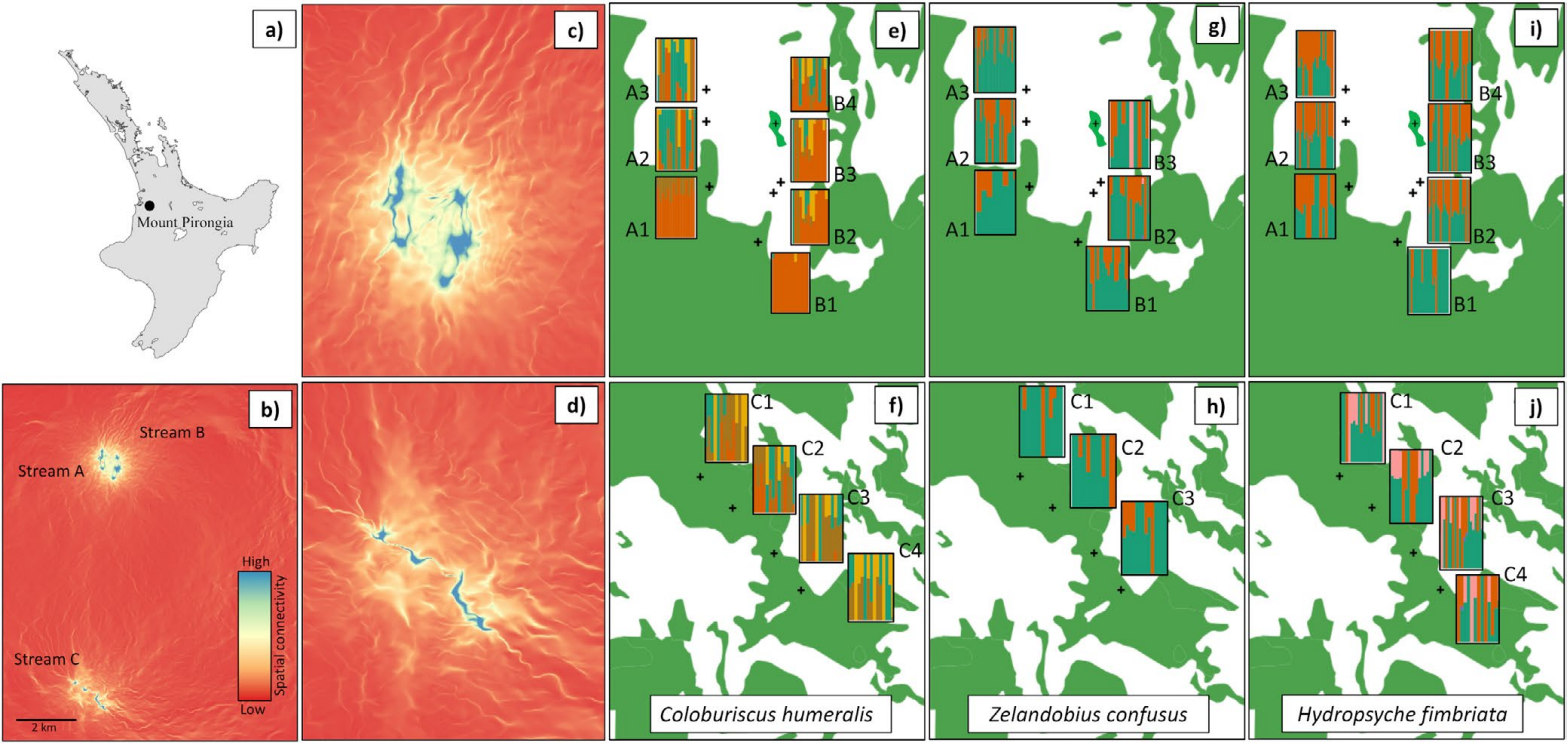
Spatial connectivity (a–d) on the left within the Mount Pirongia study area based on topographic complexity calculated from a digital elevation model. Assignments of fastSTRUCTURE genetic clusters found for the 11 sampling sites for each of the studied species are shown in panels (e–j) on the right, with land cover type coloured with: Green (forest) and white (pasture).

fastSTRUCTURE analysis suggested that *K* values of 3 and 6 were most likely (for maximising marginal likelihood and explaining structure in the data, respectively; Figure 4). For *K* = 3, only individuals from Taranaki were assigned to a distinct population, and admixture between the remaining genetic clusters was observed across Pirongia and Karioi sites. For *K* = 6, assignment of individuals to clusters was mainly region‐specific, separating populations of Pirongia, Karioi, and Taranaki (Figure 4). Within Pirongia, clusters were not exclusive to sampling sites. For Streams A and B, lower admixture between clusters was found for the source forest sites A1 and B1, and admixture increased for the remaining downstream sites within both streams (Figure 5). These results were consistent with the PCoA, showing separate groups for A1 and B1 sampling sites. Increased admixture between genetic clusters was also observed for sampling sites within the fully forested Stream C when compared to the fragmented Streams A and B.

#### 3.1.2 Zelandobius confusus

COI sequence data were analysed for a total of 114 individuals of *Z. confusus* . A total of 14 haplotypes was identified, with three haplotypes common in the network (Figure 2). The central haplotype (H1) was shared among the three different mountain regions, while the dominant haplotype (H2) was separated from H1 by three mutational steps and was found only at Pirongia. The third most frequent haplotype (H3) was found in individuals from Karioi and Pirongia and was separated from H1 by four mutational steps. Most of the remaining haplotypes split off from H1 and were found in a few or single individuals. Global *F*_ST_ revealed significant genetic differentiation among all sampling sites (0.102, *p* < 0.01). AMOVA analysis showed significant genetic differentiation when comparing populations among mountain regions (*F*_ST_ = 0.214, *p* < 0.05; Table 1). Within Pirongia, the variation between populations at the remaining hierarchical levels was non‐significant (Table 1). Mantel tests across all sampling sites showed a significant correlation between Euclidean distance and genetic differentiation (*r* = 0.588, *p* = 0.005; Figure S1), indicating IBD.

The filtered SNP data set consisted of 6388 SNPs (2.48% missing data) and 160 individuals (Table S3). AMOVA analysis revealed low but significant genetic differentiation among all sampling sites (*F*_ST_ = 0.048, *p* < 0.001, 4.8% variance). Across different geographic hierarchies, significant differentiation was observed among mountain regions, as reported for COI, but also between neighbouring catchments (*F*_ST_ = 0.016, *p* < 0.05 and *F*_ST_ = 0.044, *p* < 0.05, respectively). Pairwise *F*_ST_ values were slightly lower than pairwise Nei's D values for most of the pairwise comparisons, but both parameters showed similar patterns of divergence across pairs of populations (Table S4). Very low but significant genetic differentiation (*F*_ST_) was observed for population comparisons separated by neighbouring catchments, consistent with the AMOVA results. The highest pairwise *F*_ST_ value was 0.104 (A1/E1), which reflected the geographic distance separating these sampling sites (170.8 km). As observed for COI, IBD was indicated within the overall study area (*r* = 0.795, *p* = 0.001).

In the PCoA, the two principal coordinates explained 2.6% and 1.7% of the total genetic variation, confirming a general pattern of low genetic differentiation between populations (Figure 2). Two main clusters of individuals were observed, separating population E1 (Taranaki) from the remaining individuals located across Pirongia. Within individual streams, PCoA analysis showed no clustering of individuals associated with the riparian land cover (Figure 3). The fastSTRUCTURE analysis suggested that *K* values of 2 and 4 maximised marginal likelihood and explained structure in the data, respectively (Figure 4). The *K* = 2 result was consistent with the PCoA results, with two distinct genetic clusters clearly assigning individuals to populations from two distinct mountain regions: Pirongia (A1 to C3) and Taranaki (E1). There was no evidence for finer spatial genetic structure, with *K* = 4 mostly showing admixture between Pirongia sampling sites. As for the PCoA, we did not observe assignment of individuals to a particular genetic cluster that was associated with the land cover type (Figure 5).

#### 3.1.3 Hydropsyche fimbriata

A total of 107 *H. fimbriata* individuals was included in the COI sequence data analysis. The haplotype network showed that most individuals were assigned to four of the 19 identified haplotypes. The most dominant haplotype (H13; *n* = 23) and haplotype H8 (*n* = 14) were shared only between the adjacent streams A and B, whereas H3 (*n* = 20) and H17 (*n* = 18) were shared among all three studied streams in Mount Pirongia (Figure 2). AMOVA showed significant genetic differentiation between neighbouring catchments (*F*_ST_ = 0.142, *p* < 0.01) and within Stream A (*F*_ST_ = 0.120, *p* < 0.05; Table 1). The Mantel test indicated IBD within the Pirongia region (*r* = 0.347, *p* = 0.010).

The filtered SNP data set consisted of 1789 SNPs (1.13% of missing data) from 290 individuals (Table S3). AMOVA analysis revealed significant genetic differentiation among all sampling sites (*F*_ST_ = 0.069, *p* < 0.01, 6.9% variance), among mountain regions (*F*_ST_ = 0.180, *p* < 0.01) and between neighbouring catchments (*F*_ST_ = 0.012, *p* < 0.05), although not within individual streams (*F*_ST_ = 0.000), contrasting the COI data for Stream A (Table 1). Pairwise *F*_ST_ values were similar to pairwise Nei's *D* values for most of the pairwise comparisons, and both parameters showed similar patterns of divergence across pairs of populations (Table S4). Very low but significant genetic differentiation (*F*_ST_) was observed for population comparisons within Pirongia. Higher *F*_ST_ was found when comparing sampling sites from distinct mountain regions. For example, the highest pairwise *F*_ST_ value was 0.238 (A1/F1), which reflected the geographic distance separating these sampling sites (170.8 km). An IBD pattern was found within the overall study area, including the three mountain regions (*r* = 0.706, *p* < 0.001).

Individuals fell into four clusters in the PCoA, where the first two principal coordinates explained 5.6% and 2.2% of the genetic variation (Figure 2). Spatial structure was only observed for individuals from Taranaki (F1), which grouped together exclusively. Within individual streams, the PCoA showed no clustering of individuals that associated with riparian land cover (Figure 3). fastSTRUCTURE analysis suggested that *K* values of three and five were most likely for maximising the marginal likelihood and explaining structure in the data, respectively (Figure 4). Individuals from Taranaki that grouped together in the PCoA were assigned to a single genetic cluster in the fastSTRUCTURE analysis. This cluster also indicated a very low proportion of admixture for a few individuals from sampling sites in Pirongia. For *K* = 3, Pirongia and Karioi shared two clusters with similar admixture levels across all sampling sites, whereas at *K* = 5 an additional frequent genetic cluster appeared that showed admixture of individuals mostly from the fully forested Stream C (Figure 5).

### 3.2 Fine‐Scale Landscape Genetic Analysis Within Pirongia Populations

Simple Mantel correlations obtained for each landscape variable and each species dataset are shown in Table 2. Mantel correlations for resistance distances based on circuit theory versus least‐cost analysis generally led to similar coefficients. The highest coefficient for the variable topography was obtained using the least‐cost method (*r* = 0.686, *p* = 0.001), while a slightly lower correlation was observed with circuit theory (*r* = 0.640, *p* = 0.001). In contrast, for the variable land cover, the highest correlation was obtained using circuit theory (*r* = 0.709, *p =* 0.0001), whereas the least‐cost method showed a lower correlation (*r* = 0.557, *p* = 0.001). Mantel correlations also revealed similar coefficient values for the tested resistance cost ratios of 2:1, 5:1, and 10:1 for the two land cover variables (vegetation and pasture). Accordingly, we constructed the remaining landscape resistance model analyses using resistance distances generated with circuit theory and chose the land cover variable showing the highest coefficient as follows: forest: pasture 2:1 for *Z. confusus* and *H. fimbriata* , and forest: pasture 5:1 for *C. humeralis* (Table 2).

**TABLE 2:** Exploratory data analysis within Pirongia sites using the Mantel test correlation coefficient (*r*) for each landscape variable representing both least‐cost and CIRCUITSCAPE resistance distances.

Species	Category	Variable	Cost values	Least‐cost		Circuitscape	
				COI	SNPs	COI	SNPs
*C. humeralis*	Topography	slope	min = 1, max = 60	0.055, *p* = 0.370	0.263, *p* = 0.0379	0.003, *p* = 0.495	0.362, *p* = 0.061
	Land cover	vegetation: pasture	2:1	0.056, *p* = 0.357	0.228, *p* = 0.067	0.148, *p* = 0.197	0.382, *p* = 0.029
			5:1	0.069, *p* = 0.360	0.246, *p* = 0.069	0.197, *p* = 0.194	0.410, *p* = 0.022
			10:1	0.087, *p* = 0.294	0.236, *p* = 0.074	0.221, *p* = 0.169	0.409 *p* = 0.033
		pasture: vegetation	2:1	0.043, *p* = 0.391	0.225, *p* = 0.051	−0.086, *p* = 0.690	−0.041, *p* = 0.575
			5:1	0.030, *p* = 0.438	0.204, *p* = 0.063	−0.196, *p* = 0.816	−0.285, *p* = 0.906
			10:1	0.016, *p* = 0.460	0.164, *p* = 0.109	−0.227, *p* = 0.848	−0.333, *p* = 0.930
*Z. confusus*	Topography	slope	min = 1, max = 60	−0.060, *p* = 0.0701	0.574, *p* = 0.001	−0.011, *p* = 0.513	0.538, *p* = 0.001
	Land cover	vegetation: pasture	2:1	−0.075, *p* = 0.754	0.543, *p* = 0.004	−0.099, *p* = 0.712	0.490, *p* = 0.003
			5:1	−0.077, *p* = 0.744	0.557, *p* = 0.001	−0.126, *p* = 0.749	0.404, *p* = 0.008
			10:1	−0.073, *p* = 0.734	0.553, *p* = 0.001	−0.146, *p* = 0.739	0.350, *p* = 0.015
		pasture: vegetation	2:1	−0.080, *p* = 0.734	0.524, *p* = 0.004	−0.051, *p* = 0.640	0.402, *p* = 0.013
			5:1	−0.096, *p* = 0.783	0.517, *p* = 0.004	−0.040, *p* = 0.572	0.138, *p* = 0.247
			10:1	−0.105, *p* = 0.801	0.500, *p* = 0.004	−0.052, *p* = 0.590	0.065, *p* = 0.414
*H. fimbriata*	Topography	slope	min = 1, max = 60	0.356, *p* = 0.009	0.686, *p* = 0.001	0.508, *p* = 0.012	0.640, *p* = 0.001
	Land cover	vegetation: pasture	2:1	0.335, *p* = 0.018	0.667, *p* = 0.001	0.417, *p* = 0.003	0.709, *p* = 0.001
			5:1	0.341, *p* = 0.012	0.673, *p* = 0.002	0.488, *p* = 0.005	0.649, *p* = 0.001
			10:1	0.325, *p* = 0.027	0.665, *p* = 0.002	0.480, *p* = 0.018	0.605, *p* = 0.001
		pasture: vegetation	2:1	0.336, *p* = 0.009	0.661, *p* = 0.002	0.001, *p* = 0.494	0.354, *p* = 0.013
			5:1	0.307, *p* = 0.014	0.640, *p* = 0.002	−0.317, *p* = 0.902	−0.086, *p* = 0.631
			10:1	0.250, *p* = 0.040	0.597, *p* = 0.003	−0.395, *p* = 0.951	−0.215, *p* = 0.835

For all three studied species, Mantel correlations in Pirongia sites showed higher coefficients when resistance distances were analysed using the SNP dataset compared to the COI dataset (Table 3). These analyses showed weak support for IBD (*r* = 0.285, *p* < 0.05) and IBR (land cover *r* = 0.410, *p* < 0.02) for *C. humeralis* , although *p*‐values were not significant after Bonferroni correction. In contrast, there was a significant relationship between each of the three variables (Euclidean distance, typography, and land cover) and genetic differentiation in both the *Z. confusus* and the *H. fimbriata* SNP datasets (all *p*‐values < 0.005). In addition, the land cover variable had a significant relationship (*r* = 0.417, *p* = 0.003) with the COI dataset for *H. fimbriata* (Table 3).

**TABLE 3:** Correlation between landscape variables and genetic differentiation in Pirongia sites using simple Mantel tests and multiple regression on distance matrices (MRM).

(a) Mantel test	*Coloburiscus humeralis* (mayfly)				*Zelandobius confusus* (stonefly)				*Hydropsyche fimbriata* (caddisfly)			
	COI		SNP		COI		SNP		COI		SNP	
	*r*	*p*	*r*	*p*	*r*	*p*	*r*	*p*	*r*	*p*	*r*	*p*
Euclidean distance (GEO)	0.104	0.213	0.285	0.043	0.000	0.803	0.675	0.002*	0.347	0.010	0.715	0.001*
Slope (TOPO)	0.003	0.495	0.362	0.061	−0.011	0.513	0.538	0.001*	0.508	0.012	0.640	0.002*
Forest: pasture (LAND)	0.197	0.194	0.410	0.022	−0.099	0.712	0.490	0.003*	0.417	0.003*	0.709	0.001*
**(b) MRM**	***R*** ^**2**^	***p***	***R*** ^**2**^	***p***	***R*** ^**2**^	***p***	***R*** ^**2**^	***p***	***R*** ^**2**^	***p***	***R*** ^**2**^	***p***
(1) Full model: TOPO + LAND + GEO	—	—	0.200	0.232	—	—	0.338	0.001*	0.300	0.119	0.513	0.002*
(2) GEO	—	—	0.052	0.064	—	—	0.279	0.004*	0.117	0.018	0.444	0.003*
(3) TOPO	—	—	0.136	0.052	—	—	0.290	0.001*	0.258	0.009	0.410	0.001*
(4) LAND	—	—	0.169	0.047	—	—	0.240	0.003*	0.221	0.004*	0.502	0.001*
(5) TOPO + GEO	—	—	0.138	0.186	—	—	0.340	0.002*	0.258	0.066	0.504	0.002*
(6) LAND + GEO	—	—	0.177	0.109	—	—	0.282	0.005*	0.243	0.045	0.511	0.002*
(7) TOPO + LAND	—	—	0.180	0.190	—	—	0.304	0.001*	0.273	0.058	0.524	0.001*

*Note:* (−) Not available. Results not shown for spurious landscape variables excluded after exploratory data analysis.

Abbreviations: GEO, geographic distance (i.e., Euclidean distance); LAND, land cover (i.e., forest versus pasture); TOPO, Topography (i.e., slope).

* Variable/model still significant after Bonferroni *p*‐value correction.

**FIGURE 6:**
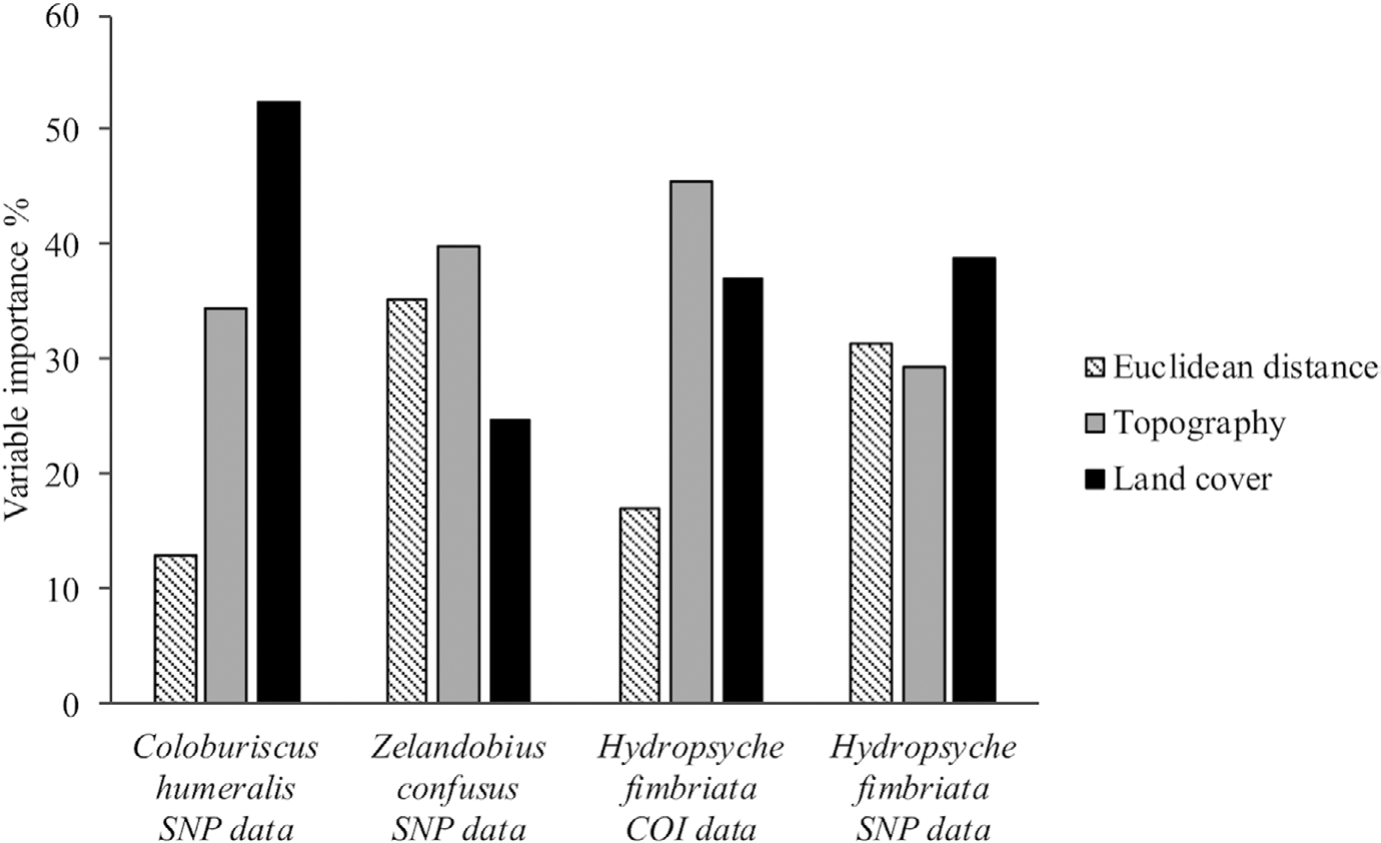
Relative variable importance for full MRM models based on hierarchical partitioning for each studied species. Variable importance is given as the percentage contribution of each variable to the overall variation explained. ^†^Results based on COI data for *Coloburiscus humeralis* (mayfly) and *Zelandobius confusus* (stonefly) are not included here, after landscape variables showed no significant influence on genetic differentiation in exploratory Mantel tests.

Results of the MRM analysis were consistent with the Mantel test results for *C. humeralis* , where a correlation between the landscape and genetic differentiation was only found for a single model using the land cover variable (*R* ^2^ = 0.169, *p* = 0.047) and was not significant after Bonferroni correction (Table 3). In *Z. confusus* , all seven candidate models showed a significant correlation (Table 3). The highest was found for the full model including all three independent variables (*R* ^2^ = 0.338, *p* = 0.001). All the candidate models also showed significant correlations with the SNP dataset for *H. fimbriata* , with the highest *R* ^2^ value found for the model combining the independent topography and land cover variables (*R* ^2^ = 0.524, *p* = 0.001). Additionally, the land cover variable showed a significant correlation with the COI genetic distances for *H. fimbriata* (*R* ^2^ = 0.221, *p* = 0.004), corroborating the Mantel test results (Table 3). Evaluating individual variable importance to the full models (Figure 6), land cover was the best predictor of genetic distances for *C. humeralis* (52% of the contribution to the model), whereas topography (39%) and Euclidean distance (35%) made important contributions to the full model for *Z. confusus* . Topography and land cover were the best predictors of genetic distance for the COI *H. fimbriata* dataset (45% and 37%, respectively), while land cover had the highest contribution (39%), followed by Euclidean distance (32%) and slope (29%) for the SNP dataset (Table 3).

## 4 Discussion

We examined functional connectivity and the influence of landscape elements on gene flow for three endemic aquatic insects within a fragmented landscape in the North Island of New Zealand. For all three species, we found a consistent pattern of genetic clustering among the three mountain regions (separated by up to ~170 km), indicating distinct population structures. Within each species, we observed varying levels of population connectivity at smaller spatial scales that may be attributed to species' dispersal abilities and distinct land cover types in their habitats.

### 4.1 Fine‐Scale Population Structure and Landscape Influence on Connectivity

The analysis conducted at fine spatial scales revealed that landscape features under IBR were more important than pure spatial factors (IBD) in predicting genetic structure across all landscape resistance scenarios examined. However, variations in the number and relative significance of these predictive landscape features were specific to each species. Similar to other studies, land‐use changes can significantly influence the abundance and aerial dispersal of aquatic insects, with the effects differing depending on the species and the habitat. For instance, Winterbourn (2007) found that montane species were more abundant in forested environments than in grasslands, while (Carlson et al. 2016) documented greater caddisfly abundance in Swedish agricultural stream systems when compared to forested habitats.

The local pattern in *C. humeralis* showed low genetic differentiation (mtDNA and SNP data), suggesting substantial population connectivity across the main study area. However, fine‐scale differentiation in the SNP dataset indicated that gene flow may not be widespread between all sampling sites, and PCoA, fastSTRUCTURE, and MRM analyses collectively suggested that land cover of the riparian zone may have a weak influence on genetic structure. Populations in fragmented Streams A and B tended to be more differentiated, whereas weaker structure was found between sites connected by riparian forest within Stream C. Thus, population connectivity was higher when the stream channel was fully covered by riparian vegetation for *C. humeralis* . For some adult aquatic insects, dispersal within the stream corridor may be preferred when the riparian zone is protected by forest, offering better habitat conditions for survival, including a cooler and moister microclimate and reduced wind speed (Collier and Smith 1997; Harding et al. 2006; Petersen et al. 2004). Combined with the potential strong flight capability of *C. humeralis* , observations during sampling showed that adults perform quick vertical movements that could facilitate flight along the stream channel, contributing to the observed genetic pattern. By contrast, the nymphs of *C. humeralis* and other mayflies tend to remain close to their natal riffle (Lancaster et al. 2011), remaining on the underside of stones, potentially limiting larval dispersal. However, fast‐flowing water and flood events increase the possibility of downstream larval dispersal, which could have contributed to the low genetic differentiation observed within the stream.

In terms of land cover, Harding et al. (2006) showed that *C. humeralis* is more predominant in continuously forested reaches and is rarely found in agricultural sites, a pattern also commonly observed in other endemic New Zealand species (Winterbourn 2007). However, the weak genetic differentiation observed in this study among adjacent fragmented streams linked via continuous open pasture may indicate that short‐distance (< 1 km) lateral dispersal across pasture occurs. This lateral movement might happen when suitable forest habitat is not found locally for *C. humeralis*, although further research is needed to test this hypothesis. In addition, environmental variables, like weather conditions, including air temperature, humidity, light intensity, and wind speed among other factors, can strongly affect flight activity in aquatic insects (Vebrová et al. 2018). Temperature, for example, may impact dispersal when fewer individuals fly outside their species' thermal windows of flight activity (Jourdan et al. 2019). Studies have also shown that the flight of aquatic beetles and heteropteran species is inhibited by increasing wind speed (Csabai and Boda 2005). Light intensity at the water surface can also help aquatic insects locate suitable habitats and oviposition sites, as has been shown for chironomids (Lerner et al. 2008). Contrasting local land cover features in our study area could also strongly affect the microclimate. For example, active dispersal may favor pathways along the stream channel, whereas passive dispersal may be more influenced by increasing wind speed in the open pasture; such factors could alternatively explain the weak genetic differentiation we identified between adjacent streams *for C. humeralis*. Based on our findings, we suggest that riparian forest cover likely facilitates connectivity among *C. humeralis* populations within the stream channel, with limited lateral dispersal across agricultural land. A more detailed analysis of the potential influences of environmental features on gene flow would help to better determine dispersal patterns in the study area for this species.

In *Z. confusus* , we found evidence for widespread gene flow among populations within the studied area, with extremely low pairwise *F*_ST_ (< 0.006) indicating high gene flow even among the most distant populations of the neighbouring catchments (~11 km). Meanwhile, subtle genetic differentiation in the SNP dataset was related to a combined effect of topography and Euclidean distance. Recently, another landscape genomics study reported high connectivity among two *Sweltsa* stonefly species across locations on both mainstem habitats and higher‐elevation tributaries in a montane ecosystem, and that the small portion of variance in allele frequency was explained by climate‐related variables indicating influence on adaptive genetic variation (Malison et al. 2022). Although stoneflies can be considered to have poor dispersal abilities, short‐distance dispersal has also been previously reported for different species (Briers et al. 2002; Sproul et al. 2014). For instance, between‐stream dispersal at small spatial scales (< 1 km) has been directly observed in mark‐recapture studies (Briers et al. 2004; MacNeale et al. 2005). Likewise, Dussex et al. (2016) found high gene flow among populations of the winged stonefly, *Zelandoperla decorata*, in different New Zealand streams separated by up to 10 km. Our findings are consistent with previous research suggesting that *Z. confusus* populations are functionally well connected within the study area and that local dispersal in this species occurs even across fragmented landscapes.

Substantial connectivity was found across most of the studied area for *H. fimbriata* , although local patterns of genetic differentiation were highly correlated with landscape elements. Topography and land cover were significantly correlated with genetic distances in both datasets, and these combined variables gave the best prediction of genetic structure for the SNP dataset. However, for both datasets, there was no marked genetic clustering that could be related to the landscape, with COI haplotypes and SNP genetic clusters closely related to one another but not shared in a geographically correlated pattern. For example, only a small number of Stream C individuals showed genetic relationships with Streams A and B; sections of steep sloping sides along stream C and/or suitable habitat provided by the local forested riparian zone could be limiting lateral dispersal in this species. Topographic effects, such as slope, have been shown to act as significant barriers to dispersal in montane mayflies, resulting in adaptive divergence, especially at higher‐elevation sites (Polato et al. 2017). Previous COI analysis of *H. fimbriata* indicated high genetic differentiation only at broad spatial scales (~100 km; Smith and Smith 2009). However, the association of this species with forested and cool streams suggests that connectivity of populations at smaller spatial scales is more likely within continuous forested habitat. Our results showed that overland dispersal by *H. fimbriata* adults from Stream C may be reduced due to local habitat features, although this does not appear to limit population connectivity more widely. Thus, the successful dispersal of *H. fimbriata* is likely to be vulnerable to further habitat fragmentation (Smith and Smith 2009).

### 4.2 Mitonuclear Discordance in *C. humeralis* and *H. fimbriata*

We observed mitonuclear discordance in two of the studied species. For *C. humeralis* , this discrepancy was strongest in the isolated Karioi population (D1), for which mtDNA data showed the highest differentiation of this from other populations, independent of the geographic distance among them. Conversely, in the SNP dataset, genetic differentiation of populations among mountain regions followed a pattern of IBD. In *H. fimbriata* , greater mtDNA versus SNP genetic differentiation occurred between populations from Pirongia. Discordance between mitochondrial and nuclear genomes has been observed in many taxa and can have a variety of causes, including *Wolbachia* infection (Smith et al. 2012; Toews and Brelsford 2012), evolutionary differences between mtDNA and nuclear markers as well as introgression and incomplete lineage sorting (Buckley et al. 2006), and sex‐mediated processes (Hurst and Jiggins 2005). No evidence of *Wolbachia* was found in BOLD trace files or the preliminary taxon ID tree, in which contaminated sequences are easily identified as a false outgroup (data not shown).

Intrinsic differences between mtDNA and nuclear markers may contribute to mitonuclear discordance. The smaller effective population size (*Ne*) of haploid mtDNA, with maternal inheritance, can increase the effect of genetic drift (Brown et al. 1979). Further, elevated mutation rates compared to nuclear genes could lead to greater differentiation (Galtier et al. 2009). For the isolated Karioi population of *C. humeralis* , this could explain the pronounced mtDNA differentiation compared to nuclear SNP markers, as drift may have had a stronger effect on mtDNA. In *H. fimbriata* , the greater mtDNA differentiation in Pirongia populations may result from the same process.

Other evolutionary processes, such as introgression (the exchange of genes between related species through hybridisation) and incomplete lineage sorting (ILS—recent gene divergence, where distinct and isolated lineages have not progressed sufficiently to allow gene sorting), could also play a role, although they generate similar genetic signatures, making them difficult to distinguish (Buckley et al. 2006). However, both processes have been shown to influence divergence between populations across a range of taxa (Dincă et al. 2019; Pavlova et al. 2013; Wang et al. 2019). Recent isolation with ILS is a possible hypothesis for the discordant results found in the *C. humeralis* Mount Karioi/D1 population. Anthropogenic deforestation in the Waikato region has resulted in isolation and fragmentation of the Pirongia Forest Park into two large, disconnected forest patches (Mount Pirongia and Mount Karioi). As a result, this fragmentation may have caused reproductive isolation between Karioi and Pirongia populations of *C. humeralis* . Coupled with the maternal inheritance and haploid nature of mtDNA, which makes lineage sorting progress faster compared to nuclear DNA (Avise 1994), ILS may explain the observed COI differentiation despite limited but ongoing nuclear gene flow for this species. Another possibility is that late Pliocene volcanic events caused historic isolation followed by subsequent contact between the Karioi and Pirongia populations for *C. humeralis* , preserving the mtDNA divergence while resulting in limited genetic differentiation in the nuclear SNPs. Historic isolation with secondary contact has been proposed as a driver of mitonuclear discordance in other aquatic insects, including the European stonefly *Dinocras cephalotes* (Elbrecht et al. 2014). Further investigation, including detailed phylogenetic and demographic analyses, is necessary to clarify the historical and contemporary processes underlying this genetic variation. Extending population comparisons to other streams in the Karioi and Pirongia regions, as well as intermediate locations between the two mountains, would provide further insights into the evolutionary patterns of *C. humeralis* .

Discordance between markers can also arise if there are differences in how selection acts on the mitochondrial genome as compared to the nuclear genome, or if there is a biased movement of either marker type driven by sex‐biased dispersal (Toews and Brelsford 2012). Despite the traditional assumption of neutrality, a number of studies have attributed mtDNA variation to natural selection (Camus et al. 2017; Galtier et al. 2009). If such selection varies geographically, then discordance between mtDNA and nuclear DNA can be expected. Sex‐biased asymmetries, such as male‐biased dispersal, can also promote gene flow for nuclear DNA in the absence of concordant movement of mtDNA (Prugnolle and de Meeus 2002), resulting in greater structure and/or narrower geographic clines for mtDNA versus nuclear DNA. Male‐biased dispersal has driven such patterns in several insect species, including bees, wasps, and ants (Bluher et al. 2020; Johnstone et al. 2012; López‐Uribe et al. 2014). In aquatic insects, this pattern has been reported, mainly for mayflies, indicating that males tend to disperse more laterally between streams and further along the stream channel than females (MacNeale et al. 2005; Petersen et al. 2004; Sabando et al. 2011; Schultheis and Hughes 2005), while the contrary has been indicated for mayflies inhabiting ponds (Caudill 2003). Our results for *H. fimbriata* indicated substantially higher genetic differentiation for COI versus SNP markers (as indicated by pairwise *F*_ST_ comparisons and AMOVA) that may be consistent with a pattern of male‐biased dispersal, as higher COI genetic differentiation was found between reaches along the stream channel, indicating females may not move as far from their natal sites. However, both markers indicated significant genetic differentiation at this small spatial scale, and we cannot currently rule out other explanations (see above). To obtain a clearer understanding of the relationship between genetic structure and dispersal capacity differences between males and females in *H. fimbriata* , direct studies of movement, such as mark‐recapture, will be necessary.

### 4.3 Implications for Conservation Management

As well as detecting differentiation over broad spatial scales, we detected species‐specific influences of landscape elements on genetic structure at finer spatial scales, suggesting different responses to habitat fragmentation in terms of dispersal and population connectivity. This result is consistent with previous studies showing that attributes affecting dispersal can vary between species and landscapes (Phillipsen et al. 2015), which are therefore not equally affected by the natural or anthropogenically driven structure of the stream habitat (Harding et al. 2006). While the three aquatic insects studied here are common in the North Island of New Zealand, each is likely affected differently by ongoing habitat fragmentation. Overall, our findings align well with previous dispersal studies on the analysed or similar species (e.g., Dussex et al. 2016; Polato et al. 2017; Smith and Smith 2009). Our work extends that knowledge by evaluating the role of landscape factors in shaping connectivity. In particular, higher dispersal capacity and lower dispersal constraints of *Z. confusus* found at small scales (< 10 km) suggest that this species may have an advantage in colonising disturbed habitats and restored sections and maintaining connectivity among proximate streams. For *C. humeralis* and *H. fimbriata* , present populations are likely to benefit most from restoration of contiguous stream reaches via riparian planting to enhance short‐distance dispersal within the stream channel. Restored sites provide suitable environmental conditions for colonisation, such as cooler temperatures and shade, thereby enhancing connectivity to source populations upstream. Based on our findings, connectivity and long‐term viability of populations could benefit from riparian/restoration planting in close proximity (< 1 km) to potential source populations in native forested sites.

Our study showed that gene flow is dominant between local populations and provides insight into how the stream's surrounding landscape features may influence species‐specific responses to habitat fragmentation, even at small spatial scales. Expanding sampling sites with a gradual geographic distance distribution between Pirongia, Karioi, and Taranaki and analysing landscape resistance across the mountain regions spatial scale would be essential for accurately distinguishing patterns of isolation by distance from other drivers of genetic structure. We caution that geographic barriers, climate events, and hydrological changes may have influenced the observed spatial genetic structure, especially at the Taranaki cluster, where samples were collected at a later stage. Longer‐term monitoring and time‐series sampling are needed to assess temporal genetic differentiation effects on genetic variations. With a broader understanding of current diversity, population connectivity, and potential barriers to gene flow/dispersal, managers can begin to make informed decisions about prioritising remaining populations and the conservation/restoration efforts that will support the successful dispersal and long‐term survival of aquatic insect populations. Our findings highlight that dispersal in similar landscapes is a complex process that is influenced by a range of environmental factors, including climate and its seasonality, water quality, predation, and competition with other species. The evolving field of landscape genetics/genomics in aquatic systems is positioned to make valuable contributions to the conservation and management of riverine ecosystems; future research should explore the interplay between these variables to obtain a comprehensive understanding of how to best maintain/restore habitat/population connectivity for aquatic insects.

### 4.4 Future Directions: Methodological Considerations in Landscape Genetics

Landscape genetics has advanced considerably over the last decade, emphasising methods to characterise functional connectivity beyond traditional assumptions, including IBD and simplified gene flow barriers. Selecting appropriate methods, resistance surface validation, and habitat heterogeneity's impact on gene flow are crucial considerations. Many studies correlate landscape resistance with genetic differentiation using Mantel tests, although their linearity assumption has been debated (Peterman and Pope 2021; Legendre et al. 2015; Meirmans 2015). While Mantel tests detect IBD under Euclidean distances, they may struggle with isolation by resistance (IBR) (Kierepka and Latch 2015). Alternative methods, such as Moran's eigenvector maps (Legendre et al. 2015) and Redundancy analysis (Meirmans 2015) have been used to improve IBR detection. Further, combining statistical tests would provide a more comprehensive landscape genetic analysis.

Developing resistance surfaces is crucial in landscape genetics, as least‐cost path (LCP) distances often explain genetic differentiation better than Euclidean distances. However, empirical data on the movement of individuals are difficult to obtain, and resistance weights frequently rely on subjective expert opinion (Zeller et al. 2012; Spear et al. 2010; Shirk et al. 2010). To improve objectivity, habitat suitability models (HSMs), or species distribution models, can generate resistance surfaces by linking species occurrence with ecological variables (Guisan et al. 2013) to better explain genetic differentiation than expert assessments (Milanesi et al. 2016; Milanesi, Holderegger, Bollmann, et al. 2017; Milanesi, Holderegger, Caniglia, et al. 2017). Applying these models to stream insects is complex due to the interplay of aquatic and terrestrial environments. High‐quality data are essential, and integrating habitat structure and dynamic environmental variables like seasonal water and temperature levels remains challenging. Studies that use three‐dimensional (3D) habitat models from Light Detection and Ranging (LiDAR) data would improve resistance surface accuracy over traditional land cover and topographic models (Milanesi, Holderegger, Bollmann, et al. 2017; Milanesi, Holderegger, Caniglia, et al. 2017) and could be used to enhance the accuracy of functional connectivity analyses for aquatic insects.

Ecological niche models (ENMs) are valuable in landscape genetics, helping identify macroecological patterns (Milanesi et al. 2018; Rolland et al. 2015). They integrate species distribution, habitat suitability, and environmental variables to create biologically informed resistance surfaces (Timoner et al. 2021). While useful for studying genetic isolation and adaptations in aquatic insects, ENMs do not explicitly model movement. Circuit theory, widely used in landscape genetics, better captures dispersal through complex environments (Manel and Holderegger 2013; Simpkins et al. 2018; Dickson et al. 2019). Integrating ENMs with circuit theory could enhance habitat and dispersal pathway identification. Recent tools like Omniscape refine connectivity modelling (Landau et al. 2021) and will require broader application in landscape genetics studies to assess their effectiveness.

## Author Contributions

**Vanessa de Araujo Barbosa:** conceptualization (equal), data curation (lead), formal analysis (lead), investigation (lead), methodology (equal), visualization (lead), writing – original draft (lead), writing – review and editing (lead). **S. Elizabeth Graham:** conceptualization (equal), formal analysis (supporting), investigation (supporting), methodology (equal), supervision (supporting), writing – review and editing (supporting). **Ian D. Hogg:** conceptualization (equal), formal analysis (supporting), methodology (supporting), supervision (supporting), writing – review and editing (supporting). **Brian J. Smith:** conceptualization (equal), formal analysis (supporting), investigation (supporting), methodology (supporting), writing – review and editing (supporting). **Angela McGaughran:** formal analysis (equal), investigation (supporting), methodology (supporting), supervision (lead), visualization (supporting), writing – review and editing (equal).

## Conflicts of Interest

The authors declare no conflicts of interest.

## Supporting information


Data S1.

## Data Availability

Genetic Data: Mitochondrial DNA sequences: Available in the Barcode of Life Datasystems (BOLD) dataset DS‐EPTNZNI a https://doi.org/10.5883/DS‐EPTNZNI and in GenBank under accession numbers OK502554–OK502876. SNP dataset: Full SNP mapping data, including unique sample identifiers that can be matched to the mtDNA data, are available in the Dryad Digital Repository at https://doi.org/10.5061/dryad.rxwdbrvm0 titled “SNP mapping scores”. Sample Metadata: Related metadata providing records for unique sample identifier tags (including georeferenced locations and period of sampling events) that can be matched to genetic data can also be found on Barcode of Life Datasystems (BOLD) dataset DS‐EPTNZNI https://doi.org/10.5883/DS‐EPTNZNI. Benefit‐Sharing Statement: We engaged with the Department of Conservation (DoC) and local councils for permission to access protected lands and collect insects in streams, with samples sent overseas for DNA sequencing. This process involved consultation with local iwi (Māori tribes), which was performed by DoC. Benefits from this research accrue from the sharing of our data and results on public databases, as described above.
