# Supplementary material for: A Landscape Genetics Approach Reveals Species‐Specific Connectivity Patterns for Stream Insects in Fragmented Habitats
Source: Ecol Evol. 2025 Mar 9;15(3):e71084. doi: 10.1002/ece3.71084 (PMC11890307; doi:10.1002/ece3.71084)
Supplement: Supplementary file 1 — Data S1. [file ECE3-15-e71084-s001.doc]

**A landscape genetics approach reveals species-specific connectivity patterns for stream insects in fragmented habitats**

Vanessa de Araujo Barbosa, S. Elizabeth Graham, Ian D. Hogg, Brian J. Smith, Angela McGaughran

Table of contents

| **Table S1** Sampling sites localities, population codes, downstream distance (from the top to the bottom sampling site) within each stream, and riparian vegetation characteristics for each sampling site. | Page 2 |
| --- | --- |
| **Table S2** Results of Mantel tests across all populations for each of the three study species using linearised *F*_ST_ as a measure of genetic distance and Euclidean distance as a measure of geographic distance. | Page 3 |
| **Table S3**. Sample sizes (*n*) of analysed data in each of the population sampling localities for the three study species. | Page 4 |
| **Table S4.** Pairwise genetic distances between sampling sites based on SNP data for each species. Upper diagonal: Nei’s *D* (Nei, 1987) and lower diagonal: *FST* (Weir & Clark Cockerham, 1984). | Page 5 |
| **Table S5.** Variance inflation factors (VIF) for each variable (i.e. distance matrix) included in the candidate Multiple Regression Models (MRMs). | Page 6 |
| **Figure S1.** Relationship between genetic (linearised *F*_ST_) and geographic (Euclidean) distances between pairs of populations for *Coloburiscus humeralis*, *Zelandobius confusus* and *Hydropsyche fimbriata*. Black dots indicate pairs of populations within Pirongia and grey dots indicate pairs of populations among mountain regions. For *H. fimbriata*, data is only available for populations within the Pirongia region. Linear trend lines indicate IBD. | Page 7 |
| **Figure S2** Elevation model and resistance matrix based on slope for the Pirongia location – main study area | Page 8 |
| **Figure S3 L**and cover resistance cost matrix based on mainland NZ land cover database | Page 9 |

**Table S1** Sampling sites localities, population codes, downstream distance (from the top to the bottom sampling site) within each stream, and riparian vegetation characteristics for each sampling site.

| Locality | Population code | Waterway distance | Riparian land cover | Coordinates |
| --- | --- | --- | --- | --- |
| Pirongia |  |  |  |  |
| Tawhitiwhiti Stream | stream A |  |  |  |
|  | A1 | 0 m | indigenous forest | 37^o^ 57' 33.0'' S, 175^o^ 05' 44.8'' E |
|  | A2 | 700 m | pasture | 37^o^ 57' 15.5'' S, 175^o^ 05' 43.9'' E |
|  | A3 | 1100 m | pasture | 37^o^ 57' 06.5'' S, 175^o^ 05' 43.9'' E |
| Te Pahu Stream | stream B |  |  |  |
|  | B1 | 0 m | indigenous forest | 37^o^ 57' 48.2'' S, 175^o^ 06' 02.1'' E |
|  | B2 | 490 m | indigenous forest fragment | 37^o^ 57' 35.0'' S, 175^o^ 06' 07.5'' E |
|  | B3 | 620 m | pasture | 37^o^ 57' 31.9'' S, 175^o^ 06' 10.4'' E |
|  | B4 | 1140 m | replanted | 37^o^ 57' 16.3'' S, 175^o^ 06' 08.1'' E |
| Ngakoaohia Stream | stream C |  |  |  |
|  | C1 | 0 m | indigenous forest | 38^o^ 03' 10.8'' S, 175^o^ 05' 02.9'' E |
|  | C2 | 550 m | indigenous forest | 38^o^ 03' 20.2'' S, 175^o^ 05' 16.9'' E |
|  | C3 | 1500 m | indigenous forest | 38^o^ 03' 35.0'' S, 175^o^ 05' 34.9'' E |
|  | C4 | 3500 m | indigenous forest | 38^o^ 03' 47.1'' S, 175^o^ 05' 46.7'' E |
| Karioi | stream D |  |  |  |
| Wainui Stream | D1 | - | indigenous forest | 37^o^ 50’ 26.1” S, 174^o^ 48’ 27.1’’E |
| Taranaki |  |  |  |  |
| Katikara Stream | stream E  E1 | - | indigenous forest | 39^o^ 12’ 07.4’’ S, 173^o^ 57’ 36.1” E |
| Patea Stream | stream F  F1 | - | indigenous forest | 39 ^o^ 19’ 26.1” S, 174 ^o^ 11’ 25.9” E |

***** All samples were collected in austral summer periods between December 2017 and January 2020.

**Table S2** Results of Mantel tests across all populations within each stream in Pirongia for each of the three study species using linearised *F*_ST_ as a measure of genetic distance and Euclidean distance as a measure of geographic distance.

|  | COI-based analysis |  | SNP-based analysis |  |
| --- | --- | --- | --- | --- |
|  | *r* | *P*-value | *r* | *P*-value |
| *Coloburiscus humeralis* (11, 11) |  |  |  |  |
| Stream A | 0.000 | 0.832 | 0.926 | 0.337 |
| Stream B | 0.589 | 0.130 | 0.637 | 0.082 |
| Stream C | 0.000 | 0.668 | 0.662 | 0.082 |
| *Zelandobius confusus* (11, 10) |  |  |  |  |
| Stream A | 0.957 | 0.114 | 0.000 | 0.834 |
| Stream B | 0.122 | 0.330 | 0.377 | 0.673 |
| Stream C | 0.000 | 0.828 | 0.903 | 0.333 |
| *Hydropsyche fimbriata* (11, 11) |  |  |  |  |
| Stream A | 0.983 | 0.168 | 0.767 | 0.336 |
| Stream B | 0.617 | 0.165 | 0.567 | 0.285 |
| Stream C | 0.767 | 0.167 | 0.516 | 0.175 |

Numbers in parentheses indicate the total number of sampling sites analysed for each species based on COI and SNP data sets, respectively. The approximate spatial scale of each stream analysed was: stream A (0.8 km), stream B (1 km), stream C (1.5 km).

**Table S3**. Sample sizes (*n*) of analysed data in each of the population sampling localities for the three study species.

| Species | Locality | COI data (*n*) | SNP data (*n*) |
| --- | --- | --- | --- |
| *Coloburiscus humeralis* | A1 | 4 | 16 |
|  | A2 | 10 | 15 |
|  | A3 | 8 | 14 |
|  | B1 | 11 | 15 |
|  | B2 | 3 | 15 |
|  | B3 | 5 | 15 |
|  | B4 | 4 | 15 |
|  | C1 | 9 | 15 |
|  | C2 | 8 | 15 |
|  | C3 | 9 | 15 |
|  | C4 | 7 | 15 |
|  | D1 | 9 | 20 |
|  | E1 | 10 | 23 |
|  | F1 | - | - |
| Total |  | 97 | 208 |
| *Zelandobius confusus* | A1 | 10 | 9 |
|  | A2 | 10 | 24 |
|  | A3 | 11 | 30 |
|  | B1 | 10 | 20 |
|  | B2 | 10 | 17 |
|  | B3 | 10 | 11 |
|  | B4 | 5 | - |
|  | C1 | 11 | 12 |
|  | C2 | 10 | 12 |
|  | C3 | 12 | 16 |
|  | C4 | 2 | - |
|  | D1 | 4 | - |
|  | E1 | 9 | 9 |
|  | F1 | - | - |
| Total |  | 114 | 160 |
| *Hydropsyche fimbriata* | A1 | **7** | 19 |
|  | A2 | 10 | 21 |
|  | A3 | 12 | 20 |
|  | B1 | 11 | 27 |
|  | B2 | 3 | 29 |
|  | B3 | 10 | 29 |
|  | B4 | 33 | 28 |
|  | C1 | 9 | 19 |
|  | C2 | 5 | 19 |
|  | C3 | 3 | 20 |
|  | C4 | 4 | 15 |
|  | D1 | - | 26 |
|  | E1 | - | 2 |
|  | F1 | - | 19 |
| Total |  | 107 | 293 |

**Table S4.** Pairwise genetic distances between sampling sites based on SNP data for each species. Upper diagonal: Nei’s *D* (Nei, 1987) and lower diagonal: *FST* (Weir & Clark Cockerham, 1984).

| *C. humeralis* | | | | | | | | | | | | | | |  |
| --- | --- | --- | --- | --- | --- | --- | --- | --- | --- | --- | --- | --- | --- | --- | --- |
|  | A1 | A2 | A3 | B1 | B2 | B3 | B4 | C1 | C2 | C3 | C4 | D1 | E1 | F1 | |
| A1 | - | 0.022 | 0.025 | 0.017 | 0.011 | 0.012 | 0.019 | 0.028 | 0.023 | 0.031 | 0.041 | 0.023 | 0.042 | - | |
| A2 | **0.038** | - | 0.009 | 0.035 | 0.018 | 0.014 | 0.013 | 0.010 | 0.012 | 0.014 | 0.016 | 0.029 | 0.040 | - | |
| A3 | **0.045** | 0.000 | - | 0.038 | 0.020 | 0.015 | 0.013 | 0.011 | 0.014 | 0.015 | 0.016 | 0.032 | 0.042 | - | |
| B1 | **0.029** | **0.074** | **0.080** | - | 0.012 | 0.018 | 0.026 | 0.043 | 0.036 | 0.047 | 0.056 | 0.027 | 0.050 | - | |
| B2 | **0.009** | **0.025** | **0.030** | **0.015** | - | 0.009 | 0.014 | 0.024 | 0.020 | 0.028 | 0.034 | 0.021 | 0.039 | - | |
| B3 | **0.012** | **0.013** | **0.016** | **0.028** | 0.001 | - | 0.011 | 0.018 | 0.016 | 0.021 | 0.027 | 0.022 | 0.038 | - | |
| B4 | **0.025** | **0.005** | **0.006** | **0.048** | **0.010** | 0.001 | - | 0.016 | 0.016 | 0.020 | 0.024 | 0.027 | 0.040 | - | |
| C1 | **0.053** | **0.004** | **0.005** | **0.094** | **0.041** | **0.024** | **0.014** | - | 0.012 | 0.011 | 0.013 | 0.036 | 0.044 | - | |
| C2 | **0.033** | 0.001 | **0.004** | **0.072** | **0.025** | **0.011** | **0.007** | 0.001 | - | 0.015 | 0.019 | 0.031 | 0.040 | - | |
| C3 | **0.056** | **0.007** | **0.008** | **0.100** | **0.044** | **0.025** | **0.019** | 0.000 | 0.003 | - | 0.016 | 0.040 | 0.047 | - | |
| C4 | **0.080** | **0.011** | **0.011** | **0.122** | **0.060** | **0.040** | **0.029** | **0.006** | **0.014** | **0.005** | - | 0.046 | 0.053 | - | |
| D1 | **0.041** | **0.054** | **0.060** | **0.057** | **0.034** | **0.037** | **0.044** | **0.072** | **0.055** | **0.076** | **0.091** | - | 0.051 | - | |
| E1 | **0.096** | **0.087** | **0.090** | **0.121** | **0.087** | **0.082** | **0.084** | **0.096** | **0.084** | **0.101** | **0.116** | **0.120** | - | - | |
| F1 | - | - | - | - | - | - | - | - | - | - | - | - | - | - | |
| *Z. confusus* | | | | | | | | | | | | | | |  |
|  | A1 | A2 | A3 | B1 | B2 | B3 | B4 | C1 | C2 | C3 | C4 | D1 | E1 | F1 | |
| A1 | - | 0.019 | 0.018 | 0.020 | 0.022 | 0.026 | NA | 0.026 | 0.025 | 0.023 | NA | NA | 0.072 | - | |
| A2 | 0.000 | - | 0.011 | 0.011 | 0.011 | 0.017 | NA | 0.018 | 0.017 | 0.014 | NA | NA | 0.063 | - | |
| A3 | 0.001 | **0.00** | - | 0.019 | 0.014 | 0.018 | NA | 0.016 | 0.015 | 0.012 | NA | NA | 0.062 | - | |
| B1 | 0.000 | 0.000 | **0.003** | - | 0.014 | 0.019 | NA | 0.018 | 0.017 | 0.015 | NA | NA | 0.063 | - | |
| B2 | 0.000 | 0.001 | **0.004** | 0.001 | - | 0.019 | NA | 0.020 | 0.020 | 0.017 | NA | NA | 0.065 | - | |
| B3 | 0.000 | 0.000 | **0.003** | 0.000 | 0.000 | - | NA | 0.025 | 0.023 | 0.021 | NA | NA | 0.070 | - | |
| B4 | NA | NA | NA | NA | NA | NA | - | NA | NA | NA | NA | NA | NA | - | |
| C1 | **0.004** | **0.005** | **0.002** | 0.003 | **0.004** | **0.005** | NA | - | 0.020 | 0.019 | NA | NA | 0.069 | - | |
| C2 | 0.002 | 0.003 | **0.001** | 0.002 | **0.003** | 0.002 | NA | 0.000 | - | 0.018 | NA | NA | 0.068 | - | |
| C3 | **0.004** | **0.003** | 0.001 | **0.003** | **0.002** | **0.002** | NA | 0.001 | 0.000 | - | NA | NA | 0.065 | - | |
| C4 | NA | NA | NA | NA | NA | NA | NA | NA | NA | NA | - | NA | NA | - | |
| D1 | NA | NA | NA | NA | NA | NA | NA | NA | NA | NA | NA | - | NA | - | |
| E1 | **0.104** | **0.097** | **0.096** | **0.097** | **0.098** | **0.101** | NA | **0.100** | **0.100** | **0.097** | NA | NA | - | - | |
| F1 | - | - | - | - | - | - | - | - | - | - | - | - | - | - | |
| *H. fimbriata* | | | | | | | | | | | | | | |  |
|  | A1 | A2 | A3 | B1 | B2 | B3 | B4 | C1 | C2 | C3 | C4 | D1 | E1 | F1 | |
| A1 | - | 0.011 | 0.013 | 0.014 | 0.011 | 0.010 | 0.011 | 0.016 | 0.015 | 0.015 | 0.018 | 0.019 | NA | 0.120 | |
| A2 | 0.000 | - | 0.012 | 0.013 | 0.010 | 0.010 | 0.011 | 0.016 | 0.014 | 0.015 | 0.018 | 0.017 | NA | 0.119 | |
| A3 | 0.001 | 0.000 | - | 0.015 | 0.011 | 0.011 | 0.011 | 0.016 | 0.014 | 0.015 | 0.017 | 0.018 | NA | 0.118 | |
| B1 | **0.009** | **0.009** | **0.011** | - | 0.011 | 0.011 | 0.014 | 0.016 | 0.016 | 0.016 | 0.020 | 0.016 | NA | 0.114 | |
| B2 | 0.002 | 0.002 | 0.002 | **0.006** | - | 0.008 | 0.008 | 0.014 | 0.013 | 0.013 | 0.015 | 0.015 | NA | 0.116 | |
| B3 | 0.001 | 0.000 | **0.002** | **0.007** | 0.000 | - | 0.008 | 0.013 | 0.012 | 0.012 | 0.015 | 0.015 | NA | 0.117 | |
| B4 | 0.003 | 0.002 | 0.001 | **0.009** | 0.000 | 0.000 | - | 0.014 | 0.014 | 0.013 | 0.016 | 0.016 | NA | 0.117 | |
| C1 | **0.011** | **0.010** | **0.009** | **0.010** | **0.008** | **0.008** | **0.009** | - | 0.012 | 0.012 | 0.015 | 0.019 | NA | 0.107 | |
| C2 | 0.007 | 0.006 | 0.003 | **0.014** | **0.006** | 0.004 | **0.008** | 0.000 | - | 0.012 | 0.014 | 0.020 | NA | 0.112 | |
| C3 | **0.009** | **0.008** | **0.006** | **0.015** | **0.006** | **0.006** | **0.007** | 0.001 | 0.000 | - | 0.014 | 0.020 | NA | 0.111 | |
| C4 | **0.012** | **0.011** | **0.006** | **0.018** | **0.007** | **0.009** | **0.010** | 0.005 | 0.000 | 0.001 | - | 0.023 | NA | 0.114 | |
| D1 | **0.020** | **0.018** | **0.019** | **0.019** | **0.163** | **0.017** | **0.019** | **0.020** | **0.022** | **0.022** | **0.027** | - | NA | 0.122 | |
| E1 | NA | NA | NA | NA | NA | NA | NA | NA | NA | NA | NA | NA | - | NA | |
| F1 | **0.238** | **0.235** | **0.233** | **0.227** | **0.227** | **0.229** | **0.230** | **0.219** | **0.226** | **0.231** | **0.231** | **0.241** | NA | - | |

Significant *FST* values *P* < 0.001 are showed in bold.

**Table S5.** Variance inflation factors (VIF) for each variable (i.e. distance matrix) included in the candidate Multiple Regression Models (MRMs).

|  | VIF values |
| --- | --- |
| Euclidean distance | 2.526 |
| Topography (slope) | 2.475 |
| Land cover (forest:pasture, 2:1) | 5.442 |
| Land cover (forest:pasture, 5:1) | 2.548 |

**Figure S1.** Relationship between genetic (linearised *F*_ST_) and geographic (Euclidean) distances between pairs of populations for *Coloburiscus humeralis*, *Zelandobius confusus* and *Hydropsyche fimbriata*. Black dots indicate pairs of populations within Pirongia and grey dots indicate pairs of populations among mountain regions. For *H. fimbriata*, data is only available for populations within the Pirongia region. Linear trend lines indicate IBD.

**Figure S2** Elevation model of Mt Pirongia and a topographic complexity raster based on the topographic slope values.

**
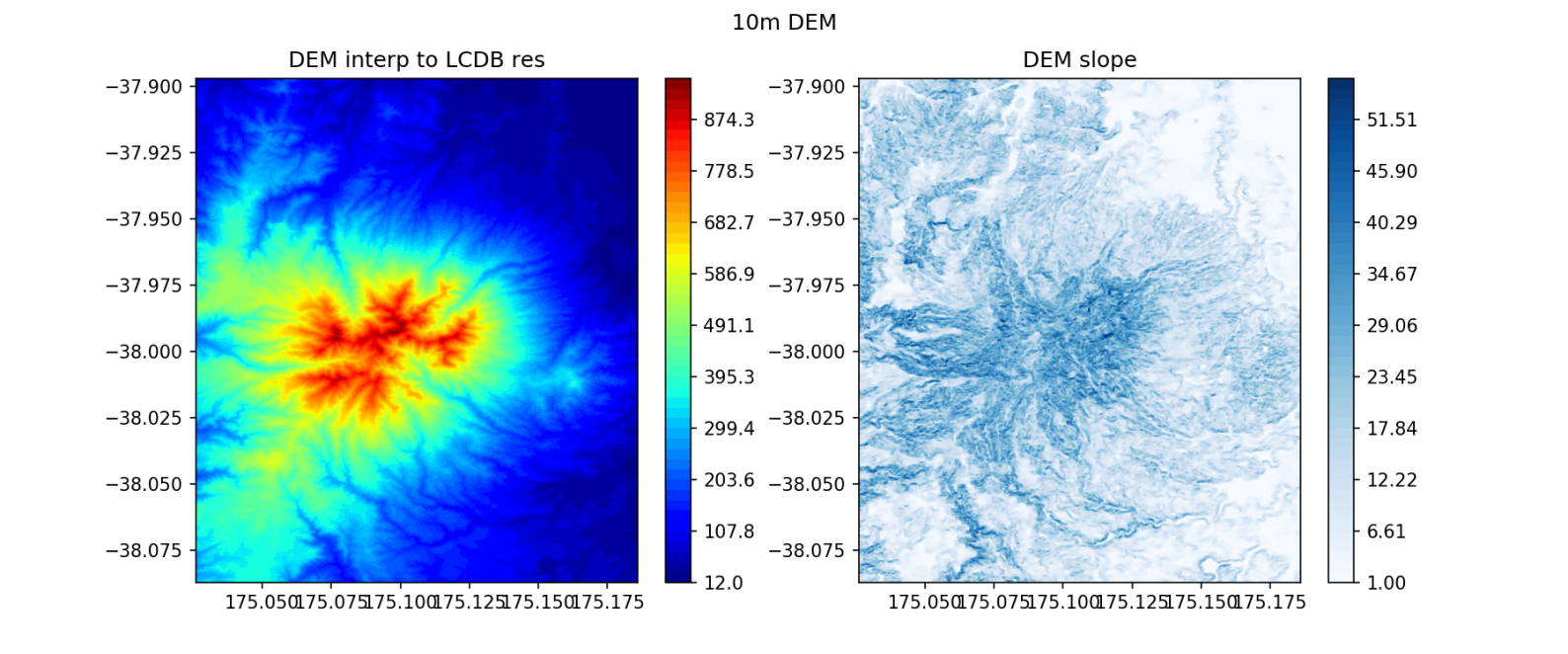
**

**Figure S3** Raster maps based on the land cover type of Mt Pirongia and surroundings. These included seven forest land cover-related classes and four grassland cover-related classes, which were used to build a final raster with ‘forest’ and ‘pasture’ classes.

**
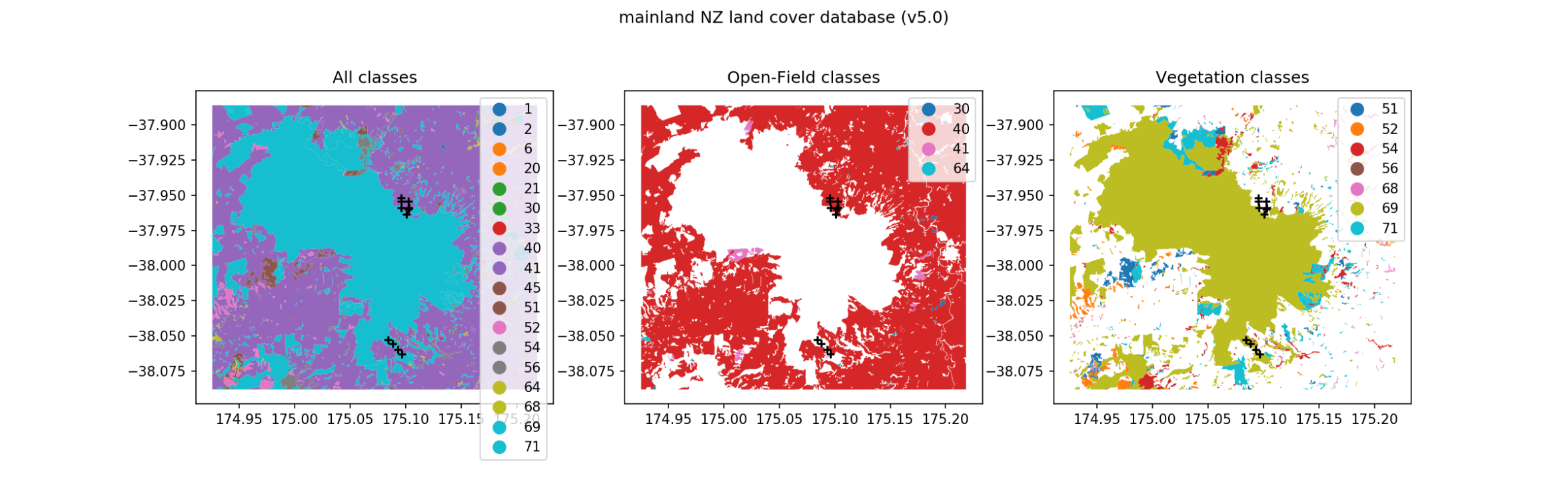
**

**
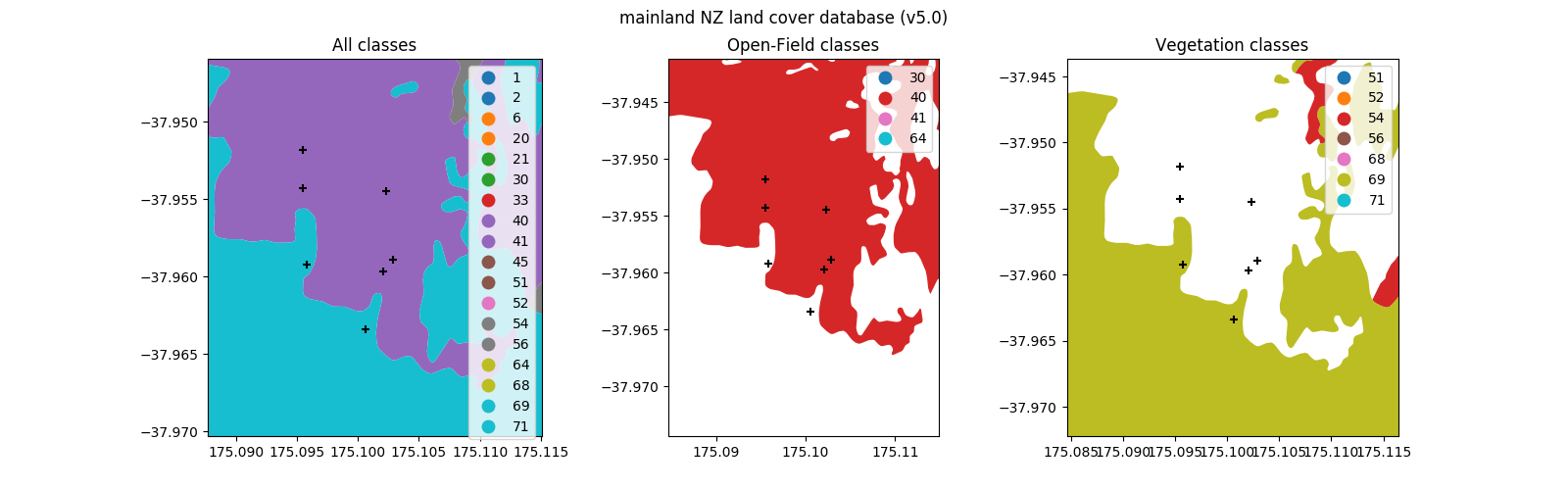

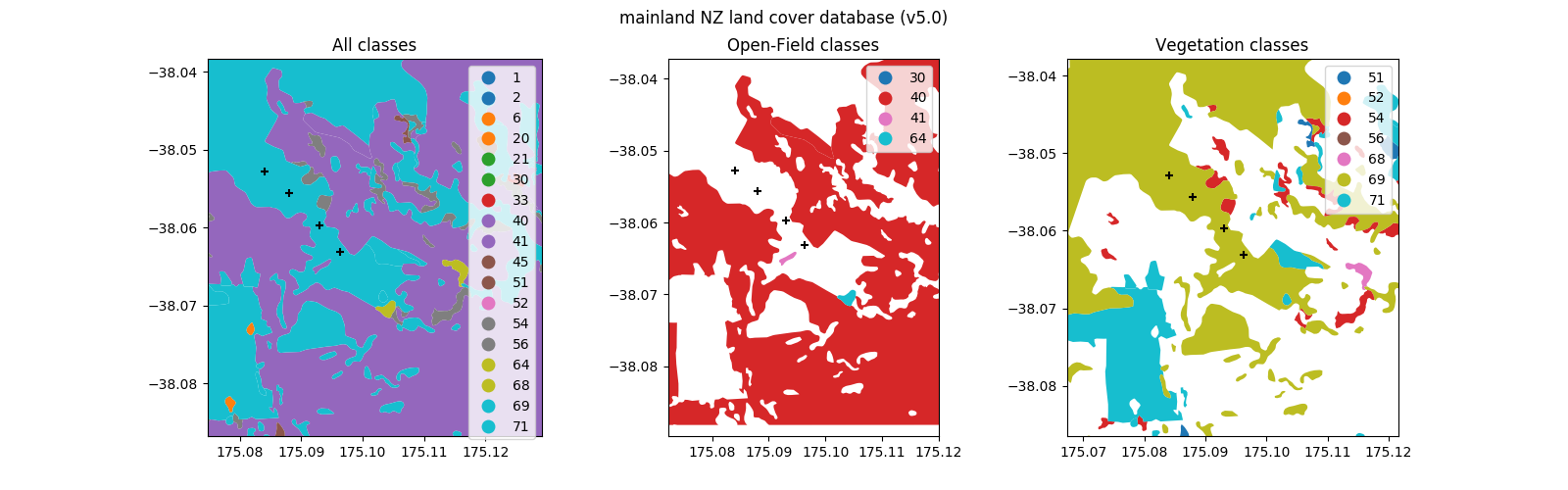
**

**
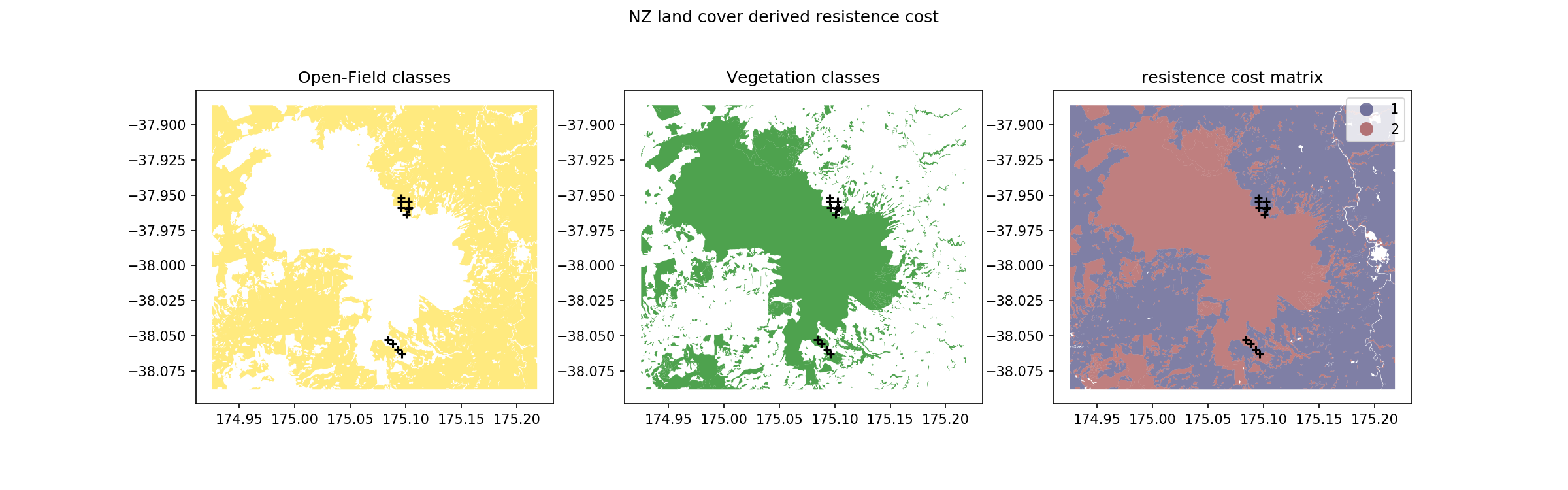
**
